# Hypoxia stimulates angiogenesis and a metabolic switch in human parathyroid adenoma cells

**DOI:** 10.1530/EO-21-0014

**Published:** 2021-07-28

**Authors:** K E Lines, M Stevenson, R Mihai, I V Grigorieva, O A Shariq, K U Gaynor, J Jeyabalan, M Javid, R V Thakker

**Affiliations:** 1 OCDEM, Radcliffe Department of Medicine, University of Oxford, Churchill Hospital, Headington, Oxford, UK; 2 Department of Endocrine Surgery, Oxford University Hospitals NHS Foundation Trust, Headington, Oxford, UK

**Keywords:** hypoxic, glycolysis, growth factor signalling, metabolic switch

## Abstract

Hypoxia, a primary stimulus for angiogenesis, is important for tumour proliferation and survival. The effects of hypoxia on parathyroid tumour cells, which may also be important for parathyroid autotransplantation in patients, are, however, unknown. We, therefore, assessed the effects of hypoxia on gene expression in parathyroid adenoma (PA) cells from patients with primary hyperparathyroidism. Cell suspensions from human PAs were cultured under normoxic or hypoxic conditions and then subjected to cDNA expression analysis. In total, 549 genes were significantly upregulated and 873 significantly downregulated. The most highly upregulated genes (carbonic anhydrase 9 (*CA9*), Solute carrier family 2A1 (*SLC2A1*) and hypoxia-inducible lipid droplet-associated protein (*HIG2*)) had known involvement in hypoxia responses. Dysregulation of oxidative phosphorylation and glycolysis pathway genes were also observed, consistent with data indicating that cells shift metabolic strategy of ATP production in hypoxic conditions and that tumour cells predominantly utilise anaerobic glycolysis for energy production. Proliferation- and angiogenesis-associated genes linked with growth factor signalling, such as mitogen-activated protein kinase kinase 1 (*MAP2K1*), Jun proto-oncogene (*JUN*) and ETS proto-oncogene 1 (*ETS1*), were increased, however, Ras association domain family member 1 (*RASSF1*), an inhibitor of proliferation was also upregulated, indicating these pathways are unlikely to be biased towards proliferation. Overall, there appeared to be a shift in growth factor signalling pathways from Jak-Stat and Ras signaling to extracellular signal-regulated kinases (ERKs) and hypoxia-inducible factor (HIF)-1α signalling. Thus, our data demonstrate that PAs, under hypoxic conditions, promote the expression of genes known to stimulate angiogenesis, as well as undergoing a metabolic switch.

## Introduction

Primary hyperparathyroidism (PHPT) is a common endocrine condition with an estimated incidence of ~50 per 100,000 in the general population (Yeh *et al.* 2013, Griebeler *et al.* 2015). The vast majority of patients with PHPT harbour a single parathyroid adenoma (PA) whose secretion of parathyroid hormone (PTH) is incompletely inhibited by hypercalcaemia (Thakker 2015). Such PAs are typically slow-growing tumours, with very low mitotic figures and low proliferative markers. Nevertheless, fragments of such tumours can readily start re-growing following inadvertent iatrogenic spillage and seeding of hyperfunctioning parathyroid tissue within the neck and/or mediastinum during parathyroid surgery, a condition known as parathyromatosis (Tublin *et al.* 2007). Furthermore, normal parathyroid glands that are removed inadvertently during total thyroidectomy and re-implanted into a muscular pocket are able to regain their vascular supply and maintain calcium homeostasis, despite initially being exposed to hypoxic conditions (Wells *et al.* 1975, Turner *et al.* 2003). However, in contrast with the ability of parathyroid tissue to maintain viability after autotransplantation *in vivo*, *in vitro* culturing of parathyroid cells is challenging, resulting in a lack of established parathyroid cell lines. A potential mechanism by which reimplantation of parathyroid tissue results in restoration of vascular supply is that parathyroid tissue can trigger spontaneous induction of angiogenesis in a vascular endothelial growth factor (VEGF)-dependent manner. Normal parathyroid glands are well vascularised to allow efficient distribution of hormones in response to external cues, such as the release of PTH in response to changes in serum calcium (Turner *et al.* 2003, Thakker 2015). Furthermore, it has been demonstrated that PA tissue is capable of inducing neovascularisation in a rabbit iris model, however, they differ from many other tumour types in the way that they very rarely undergo malignant transformation and metastasize (Saxe 1984). Therefore, the mechanisms regulating angiogenesis in parathyroid tissue and tumourigenesis are likely to be highly regulated (Turner *et al.* 2003).

The formation of new blood vessels from existing microvasculature, or angiogenesis, is stimulated by changes in the balance of pro- and anti-angiogenic factors from tumour cells, endothelial cells, macrophages, fibroblasts and the extracellular matrix, and metabolic factors, such as hypoxia. In physiological states, angiogenesis is detected in the ovary and uterus during the menstrual cycle, in the placenta during embryogenesis and during wound healing, but it is also implicated in tumour pathology (Segiet *et al.* 2015). Tumour hypoxia can occur as a result of a tumour outgrowing its blood supply or by increased oxygen consumption by rapidly proliferating cells (Kunz & Ibrahim 2003). Tumour cells can overcome the reduction in oxygen levels, continue to proliferate, and metastasize by altering their metabolism and inducing angiogenesis, a process known as the angiogenic switch (Hanahan & Folkman 1996, Kunz & Ibrahim 2003, Folkman 2006). One of the key pro-angiogenic factors involved in the stimulation of angiogenesis by parathyroid tissue is VEGF (Carter *et al.* 2000); however, the precise mechanisms behind the angiogenic switch in both normal parathyroid glands and PA cells remain to be elucidated. Human parathyroid tissue and isolated parathyroid cells were both demonstrated to increase rat microvessel growth in an *in vitro* co-culture system, and treatment of hypoparathyroidism patients with PTH 1-84 can increase serum VEGF expression, and, therefore, promote angiogenesis (Carter *et al.* 2000, Cusano *et al.* 2014). In addition, it has also been demonstrated that the glycoprotein angiopoietin 2 (AP-2), which is a mediator of VEGF-dependent angiogenesis, is stimulated in parathyroid tissue explants, and sequestering AP-2 with the endothelial cell surface receptor tyrosine kinase with Ig and EGF homology domains (Tie2) delayed the induction of angiogenesis in rat microvessel angiogenesis assays (Carter & Ward 2001).

In order to better understand the mechanisms of angiogenesis in parathyroid tissue, we isolated primary parathyroid cells from PAs obtained from patients undergoing surgery for PHPT and analysed the changes in gene expression between cells incubated in conditions of hypoxia versus normoxia. We show that, in primary human PA cells, hypoxia causes glycolysis-associated genes to be upregulated, as well as mediates a shift in growth factor signalling pathways from Jak-Stat and Ras signaling, to extracellular signal-regulated kinases (ERKs) and hypoxia-inducible factor (HIF)-1α.

## Methods

### Patient samples

Written informed consent using protocols approved by the local research ethics committee (MREC/02/2/93) was obtained from 15 patients (5 males and 10 females, age range 30–76 years) with sporadic PHPT (Table 1), in whom a decision to proceed with parathyroidectomy had been made. The PHPT in all 15 patients resulted from a single benign PA (Table 1), with none of the parathyroid tumours having evidence of multi-gland hyperplasia or malignancy. Pre-operative serum calcium and PTH concentration values were available from 13 and 12 patients, respectively. These revealed that the PHPT was associated with mild hypercalcaemia (<3.00 mmol/L) in 10 patients, moderate hypercalcaemia (>3.00–3.50 mmol/L) in 2 patients, and severe hypercalcaemia (> 3.50 mmol/L) in 1 patient, that occurred with elevated or inappropriately normal PTH concentrations in 10 and 2 patients, respectively (Table 1). Following parathyroidectomy, the sizes of the PAs were immediately recorded (range = 8–35 mm) (Table 1), and portions of the adenomas considered to be surplus to clinical needs (i.e. not necessary for confirmation of the histological diagnosis for each patient) were placed in sterile culture medium and transferred to the laboratory. Post-operative data were available from 10 patients (follow-up period = 1.5–49.8 months) and 9 were normocalcaemic and 1 had hypocalcaemia (Table 1); circulating PTH concentrations were not assessed because none of the patients had hypercalcaemia.

**Table 1:** Clinical details of patients with primary hyperparathyroidism. PA samples were obtained from 15 patients undergoing surgery for sporadic primary hyperparathyroidism due to a single PA. Samples A1–5 were used for initial cDNA expression analysis and samples V1–10 were used as a validation set for qRT-PCR and Western blot analysis.

Sample	Age	Sex	Serum calcium preop^a^ (mmol/L)	Serum intact PTH preop^b^ (pmol/L)	Sestamibi +ve	Tumour size (mm)	Most recent serum calcium^a^ (mmol/L)	Follow up (months)
A1	30	M	3.52	124.5	Not done	–	2.22	2.2
A2	57	F	2.93	13.3	Positive	35	2.46	2.7
A3	51	M	2.65	190	Not done	–	2.24	49.8
A4	71	F	2.52	16.4	Positive	15	2.4	1.5
A5	74	F	2.66	52.3	Not done	15	1.95	11.5
V1	49	F	2.59	18.3	Positive	–	2.14	16.5
V2	41	M	2.82	22.7	Positive	8	–	–
V3	57	F	3.07	25.5	Positive	11	2.33	13.7
V4	74	M	2.7	15.8	Positive	15	–	–
V5	66	F	2.81	–	Positive	12	–	–
V6	76	F	3.10	25.5	Positive	10	2.48	9.5
V7	54	M	–	6.5	Positive	15	–	–
V8	66	F	2.68	–	Positive	10	2.35	1.7
V9	63	F	–	–	Positive	12	2.55	1.6
V10	49	F	2.84	5.5	Positive	9	–	**–**

Serum calcium values shown are albumin-adjusted total calcium concentrations. ^a^normal range 2.20–2.50 mmol/L; ^b^normal range 1.6–7.2 pmol/L. PTH, parathyroid hormone.

### Cell culture

PA fragments were processed by mechanical disruption of the tissue and digestion with 2 μg/mL collagenase type II (Sigma) and 0.5 μg/mL DNAase (Invitrogen), followed by filtration through a 100 µm nylon mesh (BD Biosciences, Oxford, UK) to achieve single-cell suspensions. Approximately 200,000 cells were plated into each well of two six-well dishes and cultured in RPMI media containing 10% foetal calf serum, penicillin/streptomycin (100 U/mL/0.1 mg/mL), 0.25 mg/mL amphotericin B (Invitrogen) for 24 h at 37°C, with 5% CO_2_ and atmospheric (21%) O_2_. After 24 h, the cells had attached to the six-well dishes, the media was removed, cells were washed in PBS and cultured in low calcium keratinocyte serum-free media (Invitrogen) supplemented with EGF and bovine pituitary extract (BPE) penicillin/streptomycin (100 U/mL/0.1 mg/mL), 0.25 mg/mL amphotericin B for 72 h. Low calcium media was used to promote the growth of parathyroid cells and reduce the number of other cell types, predominantly fibroblasts. To examine the effects of hypoxia on the cell cultures, after the initial 72 h culture, one plate was transferred to a hypoxic incubator (37°C, 5% CO_2_, 1% O_2_), while the second plate remained in the normoxic incubator (37°C, 5% CO_2_, 21% O_2_), and cells cultured for a further 48 h. Of note, cells were examined microscopically pre- and post-hypoxic incubation, and no gross changes in cell number or morphology were observed. Images were captured on an Eclipse E400 (Nikon Instruments) microscope and NIS-Elements software (Nikon Instruments).

### Reverse transcription polymerase chain reaction (RT-PCR)

RNA (1 µg) extracted from cultured PA cells was converted to cDNA using the Quantitect RT Kit (Qiagen) and utilised in RT-PCRs with primers purchased from Qiagen or Sigma. For detecting expression of the parathyroid-specific genes *PTH*, *CASR* transcriptional factor glial cells missing homolog 2 (*GCM2*) and GATA-binding protein 3 (*GATA3*) custom primers (PTH–forward primer 3’-CAGCTACTAACATACCTGAACG and reverse primer 3’-GCTTCTTACGCAGCCATTCTA; CASR forward primer–3’-GACCCCTTACATAGATTACACGC and reverse primer 3’-CTCCACAGGATTTTCTCCTCG; *GCM2*–forward primer 3’-GGCATGCCCTAACTGTCATTCTGC, and reverse primer 3’-CCAGAAGACTTTGATATAGTTACTG; and *GATA3*–forward primer 3’-AGATGGCACGGGACACTACC and reverse primer 3’-GTGGTGGTCTGACAGTTCGC) were used in PCRs that utilised 2.5 μL cDNA in a 20 μL reaction, as previously described (Grigorieva *et al.* 2010); GAPDH was used as a control. For quantitative RT-PCRs (qRT-PCRs) comparing hypoxic vs normoxic incubated cells, 1 μL cDNA was used in a 10 μL reaction using the Quantitect SYBR green kit (Qiagen), as previously described (Lines *et al.* 2020), and the QuantiTect primers: SLC2A1 (QT00068957), JUN1 (QT00242956); ETS1 (QT00049133), MAP2K1 (QT00026334), RASSF1 (QT01016134) that detects transcript variants A, B, C, D and H, STAT1 (QT00074123), and CCND1 (QT00495285). For each set of cDNA samples, the house keeping gene beta-2-microglobulin (B2M) (expression of which was confirmed to be unaffected by hypoxic incubation in our gene expression profiling experiment) was quantified as a control to allow normalisation of gene expression; the relative expression of target cDNA in all qRT-PCR studies was determined using the Pfaffl method (Pfaffl 2001). Data were analysed using one-way ANOVA correcting for multiple comparisons, as previously described (Lines *et al.* 2020).

### cDNA expression array

RNA was extracted from PA cells subjected to hypoxic or normoxic conditions, using the RNeasy mini kit (Qiagen) and utilised to generate cDNAs (Quantitect RT kit (Qiagen)), and gene expression analysis was performed by hybridisation to the WG-6 v3 expression bead chip array (Illumina, Cambridge, UK). Biotin and cy3 hybridisation were used as controls. To identify significantly altered genes an asymptotic paired, T-test was undertaken within the open source R statistical environment, with a corrected *P*-value of *P* < 0.05 and a 1.5-fold dysregulation used as cut offs. Expression array data have been deposited in the National Center for Biotechnology Information’s Gene Expression Omnibus (http://www.ncbi.nlm.nih.gov/geo) database. Functional classifications were analysed using the online gene classification tool Panther (http://www.pantherdb.org) and pathway analysis was performed using Ingenuity software (http://www.ingenuity.com).

### Results

#### Expression of parathyroid cell markers in primary PA cell cultures

**Figure 1:**
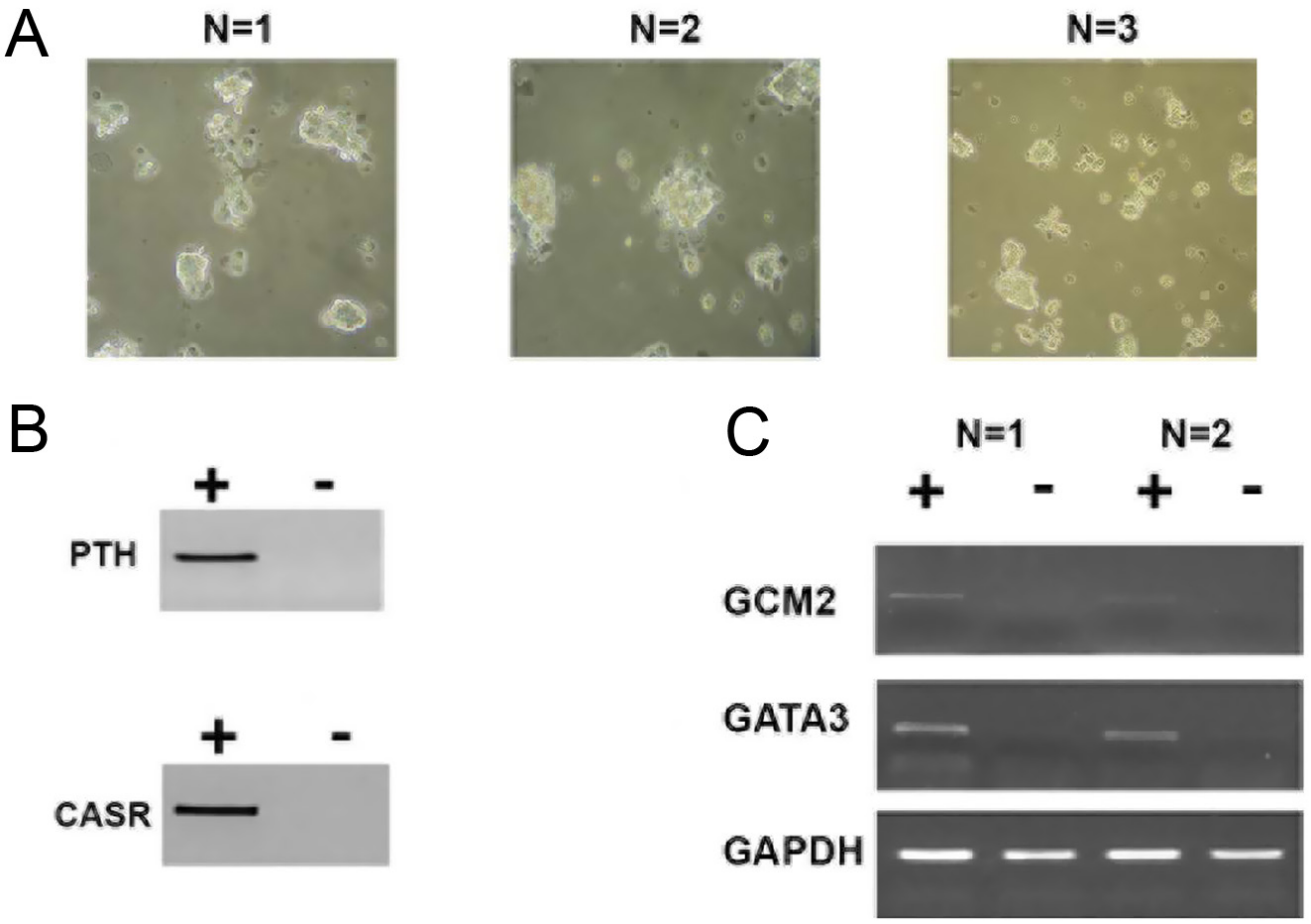
Confirmation of parathyroid cell markers. Parathyroid cells were cultured from primary PA tissue, following surgery. (A) Light microscopy images indicating the morphology of cells from three different PAs after 7 days of culture. (B) Expression of the parathyroid cell markers PTH and CaSR were confirmed on cell preparations cultured for 7 days using RT-PCR. (C) Expression of the parathyroid cell markers GATA3 and GCM2 in two different PA cell cultures was also confirmed by RT-PCR after 7 days of culture. GAPDH was used as a control. +, reverse transcriptase present; −, a negative control with no reverse transcriptase present.

To ensure primary cultures obtained from human PA tissue contained predominantly parathyroid cells, and not other cell types, including fibroblasts, cells were examined microscopically after 7 days of culture (Fig. 1A). All observed cells were rounded and there did not appear to be a high number of flat, spindle-shaped fibroblast cells. Expression of known parathyroid cell markers was also examined using RT-PCRs. Following dissociation, cultured cells retained expression of the parathyroid cell markers *PTH*, *CASR*, *GCM2*, and *GATA3* (Fig. 1B and C) for up to 7 days, thereby confirming that they maintained the characteristics of their tissue of origin.

#### Gene expression changes in primary parathyroid cells after hypoxic incubation

The effects of hypoxia on gene expression in primary human PA cultures were initially evaluated by an analysis of adenoma cells from five patients, followed by validation in cultured PA cells from 10 other patients (Table 1). Analysis of cDNA alterations in the initial set of primary PA cultures from five patients revealed that a total of 1422 genes had significant alterations of >1.5-fold (*P* < 0.05), when comparing gene expression in cells maintained in hypoxic versus normoxic conditions, with 549 of these genes being upregulated and 873 being downregulated (Supplementary Table 1, see section on supplementary materials given at the end of this article). Of note, no significant alteration in the expression of the parathyroid cell markers *PTH*, *CASR*, *GCM2* or *GATA3* was observed, however, a signal could be detected for each of these genes in all 10 samples examined, indicating they were still expressed. The 8 most highly upregulated genes carbonic anhydrase (*CA9*, 9-fold), solute carrier family 2A1 (*SLC2A1*, 4.9-fold), Enolase 2 (*ENO2*, 4.6-fold), endoplasmic reticulum oxidoreductase 1 (*ERO1L*, 4.5-fold), insulin-like growth factor-binding protein 3 (*IGFBP3*, 4.4-fold), angiopoietin-like 4 (*ANGPTL4*, 4.1-fold), hypoxia-inducible lipid droplet-associated protein (*HIG2*, 3.9-fold) and BCL interacting protein 3 (*BNIP3*, 3.9-fold) are known to be associated with responses to hypoxia (Table 2), thereby confirming that the cells are responding to the change in oxygen in their environment. Functional, bioinformatic analysis of the genes dysregulated in response to hypoxia showed that 10/11 genes associated with oxidative phosphorylation or the tricarboxylic acid cycle (TCA) cycle, (including F-type ATPases and NADH dehydrogenase) were downregulated (−1.5–2.5-fold), whereas 13 genes involved in glycolysis were upregulated (Table 3). Of the 13 genes associated with glycolysis that were significantly upregulated, the most highly upregulated were glyceraldehyde-3-phosphate dehydrogenase (*GAPDH*, 2.8-fold), aldolase fructose-bisphosphate A (*ALDOA*, 2.2-fold) and aldolase fructose-bisphosphate C (*ALDOC*, 2.6-fold) (Table 3), thereby indicating that parathyroid cells may switch their method of ATP production from oxidative phosphorylation to anaerobic glycolysis in order to survive in low oxygen concentrations. Incubation of PA cells in hypoxia also resulted in changes in the expression of genes implicated in tumourigenesis, including those involved in angiogenesis and cell proliferation. In total, 9 angiogenesis-associated genes were dysregulated, of which 8 were significantly upregulated (*SLC2A1* (4.9-fold), protein kinase C theta (*PRKCQ*, 2.4-fold), Jun proto-oncogene (*JUN*, 2.2-fold), ETS proto-oncogene (*ETS1*, 2.2-fold), mitogen-activated protein kinase kinase 1 (*MAP2K1*, 1.9-fold), recombination signal binding protein for immunoglobulin kappa J region (*RBPJ*, 1.58-fold), CRK like proto-oncogene adaptor protein (*CRKL*, 1.6-fold) and fibroblast growth factor receptor substrate 2 (*FRS2*, 1.5-fold)), and one signal transducer and activator of transcription 1 (*STAT1*) was significantly downregulated by −2.0-fold (Table 4). Of note, no changes were observed in *VEGF* expression.

**Table 2:** Hypoxia-associated genes dysregulated after PA cell culture in hypoxic vs normoxic conditions. The most highly significantly upregulated genes, as determined by fold change, were those known to be associated with responses to hypoxia, *P* < 0.05.

Gene	Fold change ((hypoxia) vs (nomoxic))
Carbonic anhydrase (*CA9*)	9.04
Solute carrier family 2A1 (*SLC2A1*)	4.94
Enolase 2 (*ENO2*)	4.57
Endoplasmic reticulum oxidoreductase 1 (*ERO1L*)	4.48
Insulin like growth factor binding protein 3 (*IGFBP3*)	4.41
Angiopoietin-like 4 (*ANGPTL4*)	4.10
Hypoxia-inducible lipid droplet-associated protein (*HIG2*)	3.93
BCL interacting protein 3 (*BNIP3*)	3.86

**Table 3:** Energy production-associated genes dysregulated after PA cell culture in hypoxic vs normoxic conditions. Genes associated with energy production pathways (according to the PANTHER gene list analysis programme) that were seen significantly dysregulated, *P* < 0.05.

Gene	Fold change
Oxidative phosphorylation genes significantly downregulated	
NAD(P)H quinone dehydrogenase 1 (*NQO1*)	−2.05
NADH:ubiquinone oxidoreductase subunit A3 (*NDUFA3*)	−1.98
NADH:ubiquinone oxidoreductase subunit B5 (*NDUFB5*)	−1.98
NADH:ubiquinone oxidoreductase subunit A2 (*NDUFA2*)	−1.89
NADH:ubiquinone oxidoreductase subunit S7 (*NDUFS7*)	−1.81
NADH:ubiquinone oxidoreductase subunit B3 (*NDUFB3*)	−1.73
NADH:ubiquinone oxidoreductase subunit S8 (*NDUFS8*)	−1.65
NADH:ubiquinone oxidoreductase subunit B6 (*NDUFB6*)	−1.65
NADH:ubiquinone oxidoreductase complex assembly factor 1 (*NDUFAF1*)	−1.64
NADH:ubiquinone oxidoreductase complex assembly factor 2 (*NDUFAF2*)	−1.55
NADH:ubiquinone oxidoreductase core subunit V2 (*NDUFV2*)	−1.55
NADH:ubiquinone oxidoreductase subunit A7 (*NDUFA7*)	−1.55
NADH:ubiquinone oxidoreductase subunit A12 (*NDUFA12*)	−1.54
NADH:ubiquinone oxidoreductase subunit AB1* (NDUFAB1*)	−1.50
Cytochrome C oxidase assembly factor heme A:farnesyltransferase COX10* (COX10*)	−1.72
Cytochrome C1 (*CYC1*)	−1.70
Succinate dehydrogenase complex subunit D (*SDHD*)	−1.77
Succinate dehydrogenase complex subunit C (*SDHC*)	−1.54
ATP synthase membrane subunit C locus 1 (*ATP5G1*)	−2.53
ATP synthase membrane subunit G (*ATP5L*)	−2.09
ATP synthase membrane subunit C locus 3 (*ATP5G3*)	−2.05
ATP synthase mitochondrial F1 complex assembly factor 1 (*ATPAF1*)	−1.68
ATP synthase peripheral stalk-membrane subunit B (*ATP5F1*)	−1.60
ATP synthase F1 subunit delta (*ATP5D*)	−1.58
ATP synthase membrane subunit F (*ATP5J2*)	−1.54
ATP synthase membrane subunit E (*ATP5I*)	−1.53
Glycolysis genes significantly upregulated	
Glyceraldehyde-3-phosphate dehydrogenase (*GAPDH*)	2.80
Aldolase, fructose-bisphosphate C (*ALDOC*)	2.59
Aldolase, fructose-bisphosphate A (*ALDOA*)	2.16
Enolase 1 (*ENO1*)	2.05

**Table 4:** Angiogenesis-associated genes dysregulated after parathyroid cell incubation in hypoxic vs normoxic conditions. Genes associated with angiogensis pathways (according to the PANTHER gene list analysis programme) that were observed to be significantly dysregulated, *P* < 0.05.

Gene	Fold change
Solute carrier family 2A1 (*SLC2A1*)	4.94
Protein kinase C theta (*PRKCQ*)	2.41
Jun proto-oncogene (*JUN*)	2.22
ETS proto-oncogene (*ETS1*)	2.15
Mitogen-activated protein kinase kinase 1 (*MAP2K1*)	1.88
Recombination signal binding protein for immunoglobulin kappa J region (*RBPJ*)	1.58
CRK like proto-oncogene adator protein (*CRKL*)	1.57
Fibroblast growth factor receptor substrate 2 (*FRS2*)	1.51
Signal transducer and activator of transcription 1 (*STAT1*)	−1.99

#### Pathway analysis of genes significantly dysregulated in primary human PA cells after hypoxic incubation

**Figure 2:**
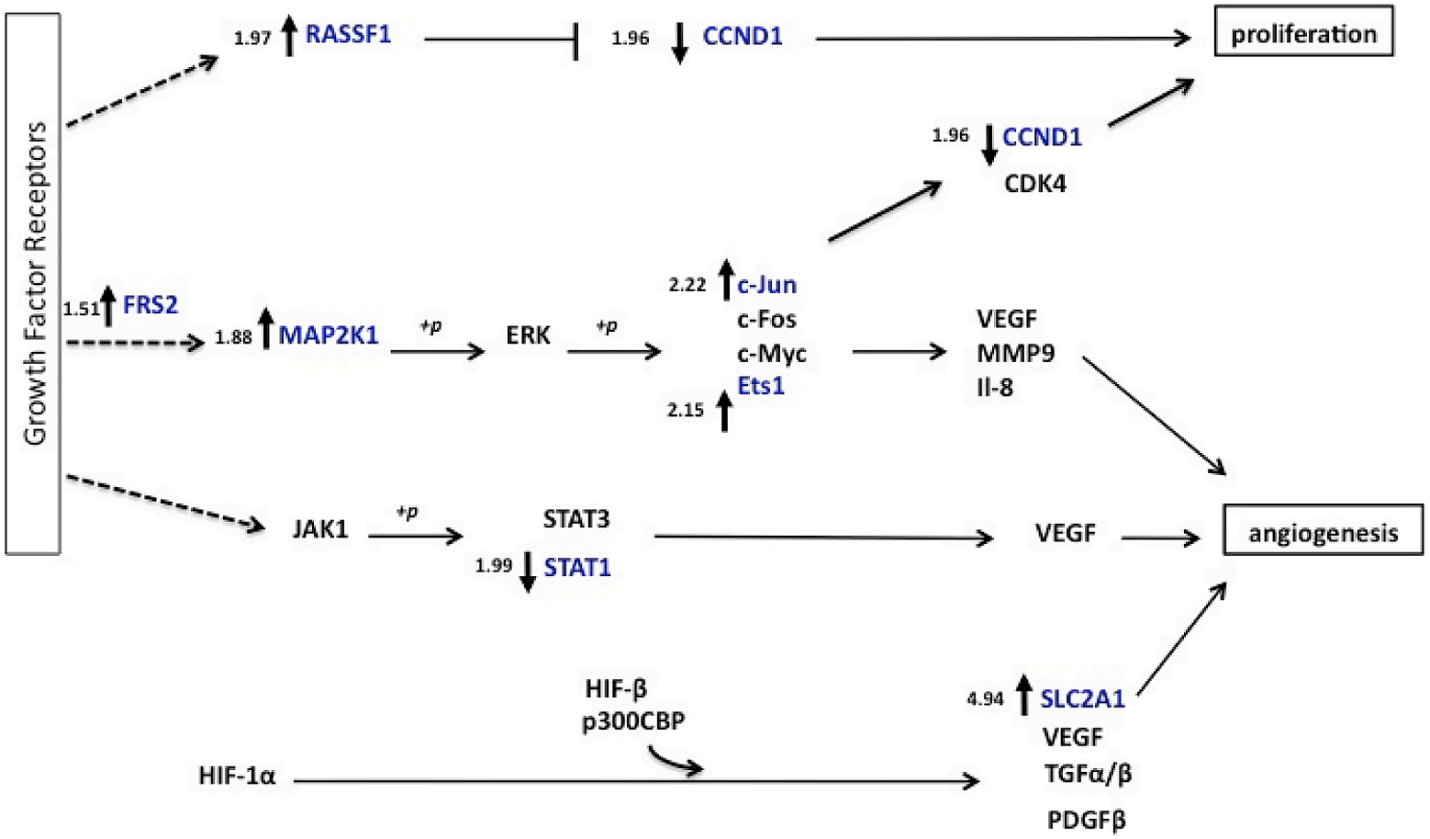
Growth factor receptor signalling pathways affected by hypoxic incubation of PA cells. Schematic diagram of growth factor receptor signalling pathways affected in PA samples after incubation in hypoxic conditions. Genes with altered expression in the cDNA expression array are indicated in blue, with the level of dysregulation indicated to the left. Dotted lines represent abbreviated signalling pathways.

Pathway analysis indicated that a number of genes significantly dysregulated in cultures from PA samples that were incubated in hypoxia are components of pro-angiogenic pathways downstream of cytokine growth factor receptors, and these included EGF receptor (*EGFR*), insulin-like growth factor 1 receptor (*IGFR*), platelet-derived growth factor receptor (*PDGFR*) and fibroblast growth factor receptor (*FGFR*), although no changes were seen in the expression of *VEGF*, which is a component of many of these pathways (Fig. 2).* MAP2K1*, *JUN* and *ETS1* levels were also significantly increased, suggesting that there may be an upregulation of ERK signalling from these receptors, whereas *STAT1* expression was significantly decreased, thereby suggesting a potential reduction in Jak-Stat signaling. Regulation of pro-angiogenic factor gene expression via hypoxia is mediated by HIF-1, a heterodimer consisting of a constitutively expressed HIF-1β subunit and oxygen and growth factor-regulated HIF-1α subunit, which in the presence of oxygen undergoes ubiquitylation and proteasomal degradation. The HIF-1-induced angiogenic pathway components (Fig. 2) include SLC2A (encoded by *SLC2A1*), c-Jun (encoded by *JUN*), ETS-1 (endoced by *ETS1*), CA9 and ALDOA, which were all upregulated by hypoxic conditions.

#### Validation of dysregulated genes in primary human PA cells after hypoxic incubation

**Figure 3:**
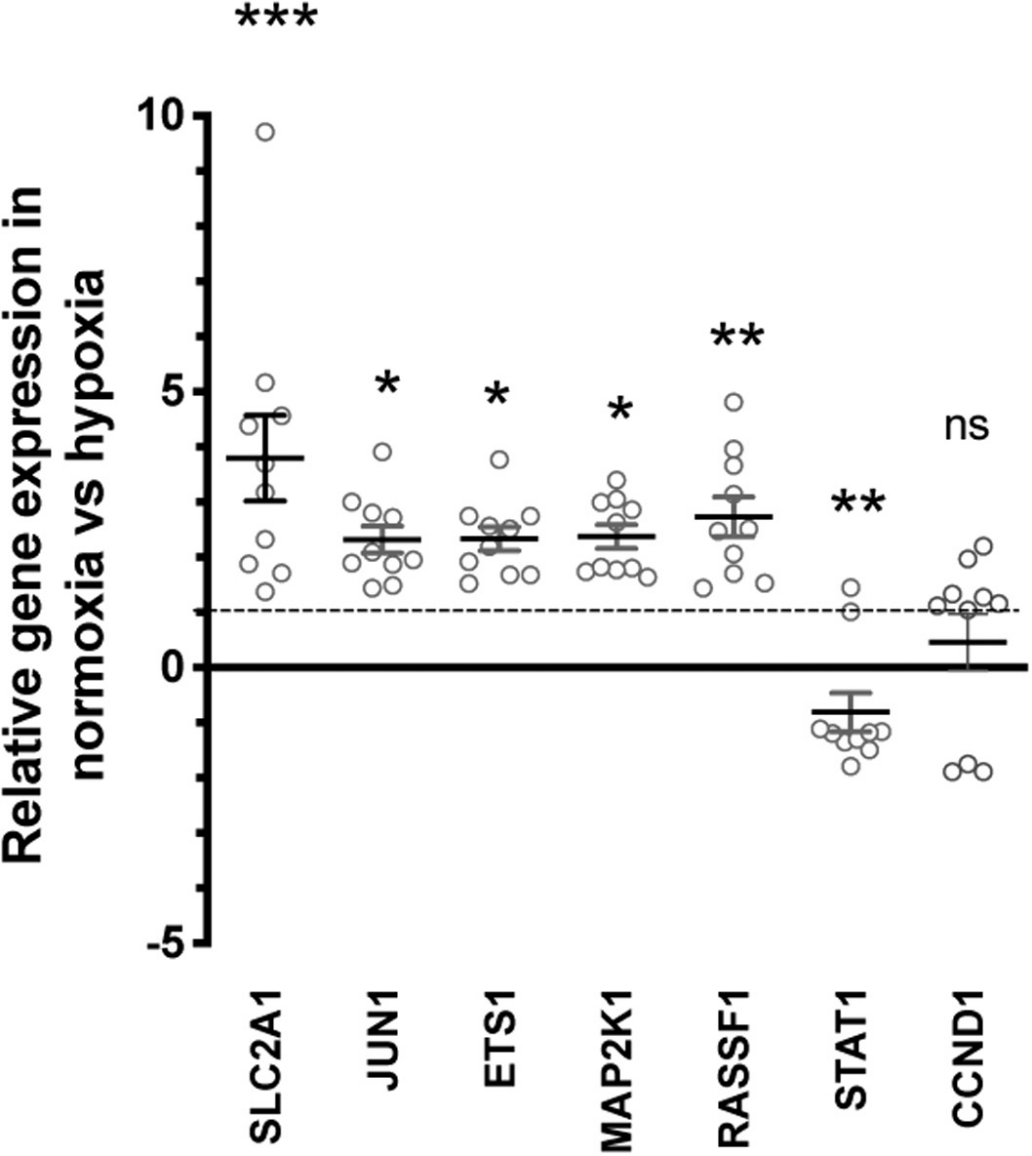
Validation of dysregulation of expression of growth factor signalling genes. Six genes known to be components of the growth factor signalling pathways were validated using quantitative RT-PCR (qRT-PCR). Relative expression between normoxic and hypoxic incubated cultures is shown, with the dotted line representing expression in normoxic conditions. B2M was used as a reference gene. Data is represented as the mean and s.e.m. of *n*  =10 biological replicates, and statistical anlasyis undertaken using a one-way ANOVA with *P < 0.05, **P < 0.005, and ***P < 0.0005.

To confirm the findings from our cDNA expression profiling, the expression of 6 dysregulated genes was examined in cultures from another 10 human primary PA samples, as part of a validation set (Table 1). As sample material was limited we focused on validating the changes in expression of genes associated with growth factor signalling pathways, as pathway analysis indicated that this signalling can result in changes in angiogenesis and cell proliferation (Fig. 2). In addition, *SLC2A1* was also included, as this gene has been associated with hypoxic and tumourigenic signalling pathways (Fig. 2). qRT-PCR analysis indicated that *CCND1* expression was variable, with no significant change observed. However, qRT-PCR analysis confirmed that *SLC2A1*, *JUN*, *ETS1*, *MAP2K1* and *RASSF1* genes were all significantly upregulated by 3.8-fold (*P* < 0.005), 2-fold (*P* < 0.005), 1.7-fold (*P* < 0.0005), 2.4-fold (*P* < 0.0005) and 2.1-fold (*P* < 0.005), respectively, and that *STAT1* was significantly downregulated by −0.8-fold (*P* < 0.0005) when incubated in hypoxic versus normoxic conditions (Fig. 3).

### Discussion

In this study, we demonstrate that primary human PA cells, when cultured in hypoxic conditions, have the ability to upregulate genes involved in anaerobic glycolysis and pro-angiogenic pathways. It has been shown previously that cells can adapt their method of ATP production under hypoxic conditions, towards anaerobic glycolysis, whereby HIF-1α promotes expression of glucose transporters and glycolytic enzymes (Agbor *et al.* 2011). Under normoxic conditions, cells utilise oxidative pathways, such as oxidative phosphorylation and the TCA cycle; however, in tumour cells, it has been shown that anaerobic glycolysis is the predominant method for energy production, in a phenomenon known as the Warburg effect (Warburg 1956, Ngo *et al.* 2015). It has been reported that this process is mediated by hypoxia-inducible factor 1 (HIF-1), which can regulate glucose transporters, including GLUT1 and GLUT3 (encoded by *SLC2A1* and *SLC2A3*, respectively) to increase flux from glucose to pyruvate, activate the pyruvate dehydrogenase kinase isozyme 1 (PDK1) to block the conversion of pyruvate to acetyl-CoA, and regulate mitochondrial biogenesis through activation of *BNIP3*, as well as suppress fatty acid β-oxidation, to control levels of reactive oxygen species and thereby sustain tumour cell survival (Zhang 2015). Little is known about the ability of PAs to change metabolic pathways under hypoxia; however, our data show that *SLC2A1* and* SLC2A3*, *PDK1* and *BNIP3* were all upregulated in PA cells cultured in hypoxic compared to normoxic conditions, thereby indicating that PAs do have the ability to switch from oxidative pathways to anaerobic glycolysis for energy production in response to hypoxia.

In addition to its role in angiogenesis, HIF proteins have been shown to have disrupted expression, including increased expression of HIF-1α and HIF-2α, in a number of cancers, and are associated with increased mortality due to a number of mechanisms including autocrine signalling, immune evasion and vascularisation (Semenza 2014). Our data suggests that in PAs there is no increase in HIF-1α or HIF-2α expression, yet there is an increase in HIF-1 signalling after hypoxic incubation, as evidenced by an increase in *GLUT1* expression, which is downstream of *HIF-1α* (Ngo *et al.* 2015). An explanation for these observations is provided by the reported increased stability in response to hypoxia of HIF-1α and HIF-2α proteins. Thus, it seems possible that hypoxia in PA cells may result in enhanced stability of HIF-1α and HIF-2α proteins leading to prolonged signalling without increases in gene expression. Moreover, this lack of increased HIF-1α or HIF-2α expression may also provide one mechanistic explanation for the predominantly benign occurrence of parathyroid tumours, which are rarely malignant and metastasise.

We also observed changes in growth factor signalling pathways in hypoxic vs normoxic conditions, including increases in expression of genes linked with vascular endothelial growth factor (VEGF) signalling, such as *MAP2K1*, *JUN* and *ETS1* (Ghosh *et al.* 2012, Koch & Claesson-Welsh 2012). Growth factor receptors, including the VEGF receptor, modulate a number of different processes, including cell proliferation, migration, differentiation and angiogenesis, through a complex network of signal transduction pathways (Koch & Claesson-Welsh 2012). These transduction pathways include RAS/RAF/MEK/ERK, JAK/STAT and PI3K/AKT, with signalling through each of these pathways leading to different biological responses (Koch & Claesson-Welsh 2012). The RAS/RAF/MEK/ERK pathway is the prominent proliferative pathway, which results in the activation of transcription factors, including the proto-oncogenes ETS-1 and c-Jun (encoded by *JUN*), which can induce the expression of genes associated with proliferation and angiogenesis (Garrett-Sinha 2013). One of the target genes of c-Jun is the cell cycle regulator cyclin D1, encoded by *CCND1* (Ouafik *et al.* 2009). In our study, we saw an increase in c-Jun expression, but in our expression profiling data, we observed a decrease in *CCND1*, which could not be confirmed in a larger set of PA samples. This may be because cyclin D1 is also negatively regulated by the tumour suppressor ras-association domain family 1, isoform A RASSF1 (Fu & Zhang 2014), which we confirmed to be significantly upregulated in hypoxic, compared to normoxic conditions. Our observed upregulation of *RASSF1* after hypoxic incubation is interesting as it has previously been reported that the *RASSF1* promoter is hypermethylated in PAs (Juhlin *et al.* 2010, Arya *et al.* 2017). Thus, our data could indicate that exposure to hypoxia may be able to reverse the inhibition of expression due to this hypermethylation. In addition to the altered expression of genes in the RAS/RAF/MEK/ERK pathway, we also observed a decrease in STAT1 expression, which is a component of the JAK/STAT signalling pathway. These findings suggest that there may be a balance between the pro-proliferative and pro-apoptotic pathways in PAs, which is biased towards angiogenesis during hypoxic conditions.

Our data indicates that parathyroid cells have the capacity to switch metabolic strategy, in response to hypoxia, by converting from oxidative phosphorylation to glycolysis, whilst upregulating angiogenesis promoting genes. Postoperative hypoparathyroidism is the most common complication after thyroidectomy and may result from inadvertent removal, devascularisation or accidental mechanical or thermal injury to normal parathyroid tissue. Parathyroid gland autotransplantation has been widely used to preserve parathyroid function, and our findings may help to elucidate the mechanisms underlying the success of this procedure. However, some studies have questioned its efficacy, with no significant decrease observed in the rates of permanent postoperative hypoparathyroidism in patients that underwent autotransplantation (Lorente-Poch *et al.* 2017). Thus, as our study only presents mRNA data, further investigation of the mechanisms highlighted by our study, particularly in confirming alterations in protein expression in autotransplaneted or parathyroid adenoma tissue using, for example, immunohistochemistry, may lead to increased understanding of the metabolic changes involved in parathyroid autotransplantation which could help to improve parathyroid graft survival and reduce the risk of permanent hypoparathyroidism in patients that undergo this procedure.

Thus, our data demonstrate that PAs under hypoxic conditions promote expression of genes known to stimulate angiogenesis and also undergo a metabolic switch from oxidative pathways to anaerobic glycolysis. This ability of parathyroid cells to switch metabolic strategy and upregulate angiogenesis-promoting genes may provide an explanation for the observed neovascularisation associated with autotransplanted PAs.

## Supplementary Material

Supplementary Table 1. Significantly dysregulated genes (p<0.05) in parathyroid adenoma cells incubated in hypoxic versus normoxic conditions.

## Declaration of interest

The authors declare that there is no conflict of interest that could be perceived as prejudicing the impartiality of the research reported.

## Funding

This work was supported by the UK Medical Research Councilhttp://dx.doi.org/10.13039/501100000265 (MRC) grant G9825289 (K E L and R V T); Royal College of Surgeons (R M); University of Oxfordhttp://dx.doi.org/10.13039/501100000769 Clarendon Scholarship and Climax Clinical Research Training Fellowship (O S); Wellcome Trusthttp://dx.doi.org/10.13039/100010269 Investigator Award (R V T); National Institute for Health Researchhttp://dx.doi.org/10.13039/100005622 (NIHR) Senior Investigator Award (R V T); and NIHR Oxford Biomedical Research Centrehttp://dx.doi.org/10.13039/501100013373 Programme (R V T).

## Author contribution statement

K E L, M S, I G, K G, J J, M J undertook experiments; R M, G S provided samples; K L, M S, R M, I G, O S, K G, J J, M J, G S, R V T were involved in writing and editing the manuscript.
