# Supplementary material for: Hypoxia stimulates angiogenesis and a metabolic switch in human parathyroid adenoma cells
Source: Endocr Oncol. 2021 Jul 28;1(1):23–32. doi: 10.1530/EO-21-0014 (PMC10265544; doi:10.1530/EO-21-0014)
Supplement: Supplementary Table 1. Significantly dysregulated genes (p<0.05) in parathyroid adenoma cells incubated in hypoxic versus normoxic conditions. [file supplementary_table_1.pdf]

**Supplementary Table 1.** Significantly dysregulated genes (p<0.05) in parathyroid adenoma cells incubated in hypoxic versus normoxic conditions.

| p-value | regulation | Fold change | Symbol | Definition |
|---|---|---|---|---|
| 0.0074 | up | 9.0403 | CA9 | carbonic anhydrase IX |
| 0.0017 | up | 4.9421 | SLC2A1 | solute carrier family 2 member 1 |
| 0.0128 | up | 4.5694 | ENO2 | enolase 2 |
| 0.0005 | up | 4.4773 | ERO1L | ERO1-like |
| 0.0203 | up | 4.4152 | IGFBP3 | insulin-like growth factor binding protein 3 transcript variant 2 |
| 0.0004 | up | 4.1034 | ANGPTL4 | angiopoietin-like 4 transcript variant 1 |
| 0.0438 | up | 3.9255 | HIG2 | hypoxia-inducible protein 2 transcript variant 1 |
| 0.0026 | up | 3.8062 | BNIP3 | BCL2/adenovirus E1B 19kDa interacting protein 3 |
| 0.0035 | up | 3.5591 | PPFIA4 | protein tyrosine phosphatase receptor type f polypeptide interacting protein alpha 4 |
| 0.0405 | up | 3.4399 | HIST2H2AA3 | histone cluster 2 H2aa3 |
| 0.0028 | up | 3.3538 | LOC644774 | PREDICTED: similar to Phosphoglycerate kinase 1 |
| 0.0414 | up | 3.3325 | HIST2H2AC | histone cluster 2 H2ac |
| 0.0038 | up | 3.3075 | PGK1 | phosphoglycerate kinase 1 |
| 0.0297 | up | 3.2986 | JAM2 | junctional adhesion molecule 2 |
| 0.0299 | up | 3.2271 | HIST2H2AA3 | histone cluster 2 H2aa3 |
| 0.0179 | up | 3.2195 | AXUD1 | AXIN1 up-regulated 1 |
| 0.0146 | up | 3.1870 | HK2 | hexokinase 2 |
| 0.0198 | up | 3.1834 | LDHA | lactate dehydrogenase A |
| 0.0076 | up | 3.1590 | TMEM45A | transmembrane protein 45A |
| 0.0006 | up | 3.1089 | PFKP | phosphofructokinase platelet |
| 0.0466 | up | 3.0865 | CXCR4 | chemokine receptor 4 transcript variant 2 |
| 0.0474 | up | 3.0794 | LOC653610 | PREDICTED: similar to Histone H2Ao |
| 0.0127 | up | 3.0430 | SLC2A3 | solute carrier family 2 member 3 |
| 0.0174 | up | 3.0361 | IGFBP5 | insulin-like growth factor binding protein 5 |
| 0.0031 | up | 3.0266 | P4HA1 | procollagen-proline 2-oxoglutarate 4-dioxygenase alpha polypeptide I |
| 0.0381 | up | 2.9729 | IGFBP3 | insulin-like growth factor binding protein 3 transcript variant 1 |
| 0.0095 | up | 2.9203 | NMB | neuromedin B transcript variant 1 |
| 0.0071 | up | 2.9198 | PCTK3 | PCTAIRE protein kinase transcript variant 1 |
| 0.0326 | up | 2.8647 | NRN1 | neuritin 1 |
| 0.0033 | up | 2.8565 | PGK1 | phosphoglycerate kinase 1 |
| 0.0120 | up | 2.8547 | HK2 | hexokinase 2 |
| 0.0000 | up | 2.8351 | ADM | adrenomedullin |
| 0.0011 | up | 2.8271 | TMOD1 | tropomodulin 1 |
| 0.0012 | up | 2.8099 | NDRG1 | N-myc downstream regulated gene 1 |
| 0.0000 | up | 2.8092 | GAPDH | glyceraldehyde-3-phosphate dehydrogenase |
| 0.0145 | up | 2.7824 | RORA | RAR-related orphan receptor A transcript variant 3 |
| 0.0259 | up | 2.7750 | MT1X | metallothionein 1X |
| 0.0001 | up | 2.7283 | GAPDH | glyceraldehyde-3-phosphate dehydrogenase |
| 0.0133 | up | 2.6895 | EGLN1 | egl nine homolog 1 |
| 0.0041 | up | 2.6747 | WDR45L | WDR45-like |
| 0.0024 | up | 2.6701 | PLOD2 | procollagen-lysine 2-oxoglutarate 5-dioxygenase 2 transcript variant 2 |
| 0.0342 | up | 2.6462 | DPYSL4 | dihydropyrimidinase-like 4 |
| 0.0313 | up | 2.6449 | LOC649270 | PREDICTED: similar to aminopeptidase puromycin sensitive transcript variant 2 |
| 0.0052 | up | 2.6373 | NFIL3 | nuclear factor interleukin 3 regulated |

| | | | | |
|---|---|---|---|---|
| 0.0030 | up | 2.6239 | ZNF395 | zinc finger protein 395 |
| 0.0016 | up | 2.5976 | GPI | glucose phosphate isomerase |
| 0.0040 | up | 2.5856 | ALDOC | aldolase C fructose-bisphosphate |
| 0.0343 | up | 2.5515 | SLC6A10P | solute carrier family 6 member 10 on chromosome 16 |
| 0.0325 | up | 2.5441 | KLF4 | Kruppel-like factor 4 |
| 0.0005 | up | 2.5352 | GBE1 | glucan branching enzyme 1 |
| 0.0190 | up | 2.5332 | TIMP3 | TIMP metallopeptidase inhibitor 3 |
| 0.0273 | up | 2.5250 | RIOK3 | RIO kinase 3 transcript variant 1 |
| 0.0265 | up | 2.5195 | IGFBP5 | insulin-like growth factor binding protein 5 |
| 0.0230 | up | 2.4927 | STC1 | stanniocalcin 1 |
| 0.0020 | up | 2.4922 | FAM80A | family with sequence similarity 80 member A |
| 0.0200 | up | 2.4889 | S100A10 | S100 calcium binding protein A10 |
| 0.0000 | up | 2.4813 | BNIP3L | BCL2/adenovirus E1B 19kDa interacting protein 3-like |
| 0.0279 | up | 2.4712 | RORA | RAR-related orphan receptor A transcript variant 3 |
| 0.0250 | up | 2.4684 | LOC441054 | PREDICTED: hypothetical protein LOC441054 |
| 0.0260 | up | 2.4460 | S100A10 | S100 calcium binding protein A10 ) |
| 0.0374 | up | 2.4382 | ZBTB43 | zinc finger and BTB domain containing 43 |
| 0.0006 | up | 2.4318 | AK3L1 | adenylate kinase 3-like 1 nuclear gene encoding mitochondrial protein transcript variant 7 |
| 0.0397 | up | 2.4295 | SLC16A3 | solute carrier family 16 member 3 transcript variant 2 |
| 0.0048 | up | 2.4159 | LOC554223 | PREDICTED: hypothetical LOC554223 transcript variant 1 misc RNA |
| 0.0201 | up | 2.4132 | PRKCQ | protein kinase C theta |
| 0.0022 | up | 2.4057 | P4HA2 | procollagen-proline 2-oxoglutarate 4-dioxygenase alpha polypeptide II transcript variant 2 |
| 0.0006 | up | 2.3964 | GAPDH | glyceraldehyde-3-phosphate dehydrogenase |
| 0.0046 | up | 2.3930 | WDR54 | WD repeat domain 54 |
| 0.0216 | up | 2.3917 | TMEM45A | transmembrane protein 45A |
| 0.0017 | up | 2.3899 | AK3L1 | adenylate kinase 3-like 1 nuclear gene encoding mitochondrial protein transcript variant 7 |
| 0.0021 | up | 2.3843 | FAM57A | family with sequence similarity 57 member A |
| 0.0054 | up | 2.3790 | | cDNA: FLJ23313 fis clone HEP11919 |
| 0.0252 | up | 2.3740 | IRF2BP2 | interferon regulatory factor 2 binding protein 2 transcript variant 1 |
| 0.0230 | up | 2.3676 | NOL3 | nucleolar protein 3 |
| 0.0006 | up | 2.3575 | FAM162A | family with sequence similarity 162 member A |
| 0.0227 | up | 2.3369 | PDK3 | pyruvate dehydrogenase kinase isozyme 3 |
| 0.0063 | up | 2.3355 | TSC22D2 | TSC22 domain family member 2 |
| 0.0070 | up | 2.3305 | SPAG4 | sperm associated antigen 4 |
| 0.0284 | up | 2.3235 | PFKFB3 | 6-phosphofructo-2-kinase/fructose-26-biphosphatase 3 |
| 0.0002 | up | 2.3197 | BNIP3L | BCL2/adenovirus E1B 19kDa interacting protein 3-like |
| 0.0032 | up | 2.3161 | CTNNAL1 | catenin alpha-like 1 |
| 0.0077 | up | 2.2855 | ERRFI1 | ERBB receptor feedback inhibitor 1 |
| 0.0300 | up | 2.2753 | AKAP12 | A kinase anchor protein 12 transcript variant 1 |
| 0.0136 | up | 2.2665 | PLOD2 | procollagen-lysine 2-oxoglutarate 5-dioxygenase 2 transcript variant 2 |
| 0.0193 | up | 2.2521 | FEM1C | fem-1 homolog c |
| 0.0091 | up | 2.2414 | CRABP2 | cellular retinoic acid binding protein 2 |
| 0.0014 | up | 2.2410 | AK3L1 | adenylate kinase 3-like 1 nuclear gene encoding mitochondrial protein transcript variant 6 |
| 0.0064 | up | 2.2410 | PGAM1 | phosphoglycerate mutase 1 |
| 0.0375 | up | 2.2363 | AMPD3 | adenosine monophosphate deaminase transcript variant 2 |
| 0.0079 | up | 2.2303 | MAFF | v-maf musculoaponeurotic fibrosarcoma oncogene homolog F transcript variant 1 |
| 0.0227 | up | 2.2277 | BHLHB2 | basic helix-loop-helix domain containing class B 2 |

| | | | | |
|---|---|---|---|---|
| 0.0103 | up | 2.2196 | JUN | jun oncogene |
| 0.0128 | up | 2.2067 | SLC15A4 | solute carrier family 15 member 4 |
| 0.0090 | up | 2.1943 | PIAS2 | protein inhibitor of activated STAT 2  transcript variant beta |
| 0.0069 | up | 2.1792 | P4HA2 | procollagen-proline 2-oxoglutarate 4-dioxygenase  alpha polypeptide II  transcript variant 3 |
| 0.0077 | up | 2.1743 | PYGL | phosphorylase glycogen liver |
| 0.0183 | up | 2.1718 | ALOXE3 | arachidonate lipoxygenase 3 |
| 0.0144 | up | 2.1669 | ERGIC1 | endoplasmic reticulum-golgi intermediate compartment  1  transcript variant 2 |
| 0.0010 | up | 2.1627 | ALDOA | aldolase A fructose-bisphosphate  transcript variant 2 |
| 0.0115 | up | 2.1603 | FER1L4 | fer-1-like 4   on chromosome 20 |
| 0.0114 | up | 2.1583 | | PREDICTED:  hypothetical LOC388214 |
| 0.0016 | up | 2.1493 | BIRC3 | baculoviral IAP repeat-containing 3  transcript variant 2 |
| 0.0040 | up | 2.1454 | ETS1 | v-ets erythroblastosis virus E26 oncogene homolog 1 |
| 0.0187 | up | 2.1261 | RTN4 | reticulon 4  transcript variant 3 |
| 0.0054 | up | 2.1205 | PPP2R5B | protein phosphatase 2 regulatory subunit B' beta isoform |
| 0.0414 | up | 2.1188 | JMJD1A | jumonji domain containing 1A |
| 0.0334 | up | 2.1150 | JMJD6 | jumonji domain containing 6  transcript variant 2 |
| 0.0398 | up | 2.1059 | C4orf31 | chromosome 4 open reading frame 31 |
| 0.0024 | up | 2.0998 | FSTL3 | follistatin-like 3 |
| 0.0003 | up | 2.0939 | ALDOA | aldolase A fructose-bisphosphate  transcript variant 3 |
| 0.0461 | up | 2.0936 | NGLY1 | N-glycanase 1 |
| 0.0061 | up | 2.0931 | RIOK3 | RIO kinase 3 |
| 0.0007 | up | 2.0917 | ZNF669 | zinc finger protein 669 |
| 0.0070 | up | 2.0896 | TSC22D2 | TSC22 domain family member 2 |
| 0.0337 | up | 2.0821 | OTUD1 | PREDICTED:  OTU domain containing 1 |
| 0.0065 | up | 2.0768 | PGAM4 | phosphoglycerate mutase family member 4 |
| 0.0256 | up | 2.0700 | EGLN3 | egl nine homolog 3 |
| 0.0184 | up | 2.0654 | MXRA7 | matrix-remodelling associated 7  transcript variant 1 |
| 0.0384 | up | 2.0626 | LOC143666 | PREDICTED:  hypothetical protein LOC143666 |
| 0.0084 | up | 2.0545 | RNF24 | ring finger protein 24 |
| 0.0020 | up | 2.0506 | CTNNAL1 | catenin  alpha-like 1 |
| 0.0030 | up | 2.0487 | ENO1 | enolase 1 |
| 0.0102 | up | 2.0384 | SERTAD2 | SERTA domain containing 2 |
| 0.0087 | up | 2.0237 | CLK1 | CDC-like kinase 1 |
| 0.0276 | up | 2.0133 | SFXN3 | sideroflexin 3 |
| 0.0349 | up | 2.0126 | C9orf25 | chromosome 9 open reading frame 25 |
| 0.0023 | up | 2.0125 | LOC123688 | similar to RIKEN cDNA C630028N24 gene |
| 0.0229 | up | 2.0114 | LOC650128 | PREDICTED:  similar to CG1486-PA isoform A transcript variant 4 |
| 0.0182 | up | 2.0101 | LOC732007 | PREDICTED:  similar to Phosphoglycerate mutase 1 |
| 0.0015 | up | 2.0100 | SYT11 | synaptotagmin XI |
| 0.0112 | up | 2.0088 | ETS1 | v-ets erythroblastosis virus E26 oncogene homolog 1 |
| 0.0056 | up | 1.9997 | ADAMTS9 | ADAM metallopeptidase with thrombospondin type 1 motif 9 |
| 0.0050 | up | 1.9958 | LOC346887 | PREDICTED:  similar to solute carrier family 16  member 14 |
| 0.0456 | up | 1.9765 | OTUD1 | PREDICTED:  OTU domain containing 1 |
| 0.0064 | up | 1.9711 | TP53BP2 | tumor protein p53 binding protein 2  transcript variant 1 |
| 0.0215 | up | 1.9706 | ISG20 | interferon stimulated exonuclease gene 20kDa |
| 0.0036 | up | 1.9701 | MLL5 | myeloid/lymphoid or mixed-lineage leukemia 5   transcript variant 1 |
| 0.0126 | up | 1.9694 | RASSF1 | Ras association  domain family member 1  transcript variant A |

| | | | | |
|---|---|---|---|---|
| 0.0087 | up | 1.9690 | CLK1 | CDC-like kinase 1  transcript variant 2 |
| 0.0292 | up | 1.9651 | PPTC7 | PTC7 protein phosphatase homolog |
| 0.0234 | up | 1.9618 | PIAS2 | protein inhibitor of activated STAT 2  transcript variant alpha |
| 0.0105 | up | 1.9615 | NRIP3 | nuclear receptor interacting protein 3 |
| 0.0329 | up | 1.9612 | LOC338758 | PREDICTED:  hypothetical protein LOC338758 |
| 0.0239 | up | 1.9593 | LOC731007 | PREDICTED:  similar to Adenylate kinase isoenzyme 4 mitochondrial |
| 0.0382 | up | 1.9587 | POL3S | polyserase 3 |
| 0.0003 | up | 1.9581 | DUSP1 | dual specificity phosphatase 1 |
| 0.0139 | up | 1.9574 | PLOD2 | procollagen-lysine 2-oxoglutarate 5-dioxygenase 2  transcript variant 1 |
| 0.0109 | up | 1.9392 | CLCNKA | chloride channel Ka  transcript variant 2 |
| 0.0026 | up | 1.9354 | PTRF | polymerase I and transcript release factor |
| 0.0186 | up | 1.9351 | CLK1 | CDC-like kinase 1 |
| 0.0090 | up | 1.9347 | TICAM1 | toll-like receptor adaptor molecule 1  transcript variant 2 |
| 0.0147 | up | 1.9336 | SERPINE1 | serpin peptidase inhibitor clade E  member 1 |
| 0.0174 | up | 1.9322 | PGAM1 | phosphoglycerate mutase 1 |
| 0.0491 | up | 1.9296 | KIAA1244 | KIAA1244 |
| 0.0012 | up | 1.9272 | ADSSL1 | adenylosuccinate synthase like 1  transcript variant 2 |
| 0.0330 | up | 1.9247 | NUAK1 | NUAK family SNF1-like kinase 1 |
| 0.0120 | up | 1.9216 | TPI1 | triosephosphate isomerase 1 |
| 0.0237 | up | 1.9199 | HRASLS | HRAS-like suppressor |
| 0.0184 | up | 1.9199 | LOC653596 | PREDICTED:  similar to RAN-binding protein 2-like 1 isoform 2 transcript variant 11 |
| 0.0027 | up | 1.9132 | SLC5A8 | solute carrier family 5  member 8 |
| 0.0059 | up | 1.8923 | MIF | macrophage migration inhibitory factor |
| 0.0367 | up | 1.8922 | CCNL1 | cyclin L1 |
| 0.0350 | up | 1.8921 | ITPKA | inositol 145-trisphosphate 3-kinase A |
| 0.0232 | up | 1.8871 | FNBP1 | formin binding protein 1 |
| 0.0146 | up | 1.8865 | LOC196752 | similar to CG32542-PA |
| 0.0066 | up | 1.8833 | RASSF1 | Ras association  domain family 1  transcript variant B |
| 0.0036 | up | 1.8832 | LOC286016 | hypothetical protein LOC286016  non-coding RNA |
| 0.0178 | up | 1.8824 | CXCR7 | chemokine  receptor 7 |
| 0.0193 | up | 1.8813 | ALS2CR13 | amyotrophic lateral sclerosis 2  chromosome region candidate 13 |
| 0.0032 | up | 1.8777 | MAP2K1 | mitogen-activated protein kinase kinase 1 |
| 0.0192 | up | 1.8774 | TMEM91 | transmembrane protein 91 |
| 0.0214 | up | 1.8773 | VKORC1 | vitamin K epoxide reductase complex subunit 1  transcript variant 1 |
| 0.0206 | up | 1.8768 | LGALS1 | lectin galactoside-binding soluble 1 |
| 0.0084 | up | 1.8755 | LOC732165 | PREDICTED:  similar to Triosephosphate isomerase   transcript variant 2 |
| 0.0487 | up | 1.8752 | | cDNA FLJ31407 fis clone NT2NE2000137 |
| 0.0342 | up | 1.8712 | PGAM4 | phosphoglycerate mutase family member 4 |
| 0.0242 | up | 1.8676 | CA5B | carbonic anhydrase VB mitochondrial  nuclear gene encoding mitochondrial protein |
| 0.0183 | up | 1.8661 | SLC35E1 | solute carrier family 35 member E1 |
| 0.0450 | up | 1.8644 | CENTG2 | centaurin gamma 2 |
| 0.0022 | up | 1.8600 | FCAR | Fc fragment of IgA receptor for  transcript variant 9 |
| 0.0016 | up | 1.8592 | MAGT1 | magnesium transporter 1 |
| 0.0189 | up | 1.8587 | | UI-E-EJ1-aka-e-09-0-UIr1 UI-E-EJ1  cDNA clone UI-E-EJ1-aka-e-09-0-UI 5  sequence |
| 0.0275 | up | 1.8566 | SH3GL1 | SH3-domain GRB2-like 1 |
| 0.0311 | up | 1.8558 | LOC649639 | PREDICTED:  hypothetical protein LOC649639 |
| 0.0300 | up | 1.8537 | RDH13 | retinol dehydrogenase 13 |

| | | | | |
|---|---|---|---|---|
| 0.0191 | up | 1.8532 | MXRA7 | matrix-remodelling associated 7 transcript variant 2 |
| 0.0049 | up | 1.8527 | PPP1R13L | protein phosphatase 1 regulatory subunit 13 like |
| 0.0174 | up | 1.8517 | PGAM4 | phosphoglycerate mutase family member 4 |
| 0.0036 | up | 1.8470 | SPTBN1 | spectrin beta non-erythrocytic 1 transcript variant 1 |
| 0.0057 | up | 1.8448 | | FNPANH10 FNP cDNA sequence |
| 0.0078 | up | 1.8441 | KIAA0363 | PREDICTED: KIAA0363 protein |
| 0.0255 | up | 1.8412 | MEF2A | myocyte enhancer factor 2A |
| 0.0109 | up | 1.8404 | SLC15A4 | solute carrier family 15 member 4 |
| 0.0133 | up | 1.8399 | CLCNKA | chloride channel Ka transcript variant 1 |
| 0.0174 | up | 1.8388 | ARRDC3 | arrestin domain containing 3 |
| 0.0001 | up | 1.8349 | FKBP14 | FK506 binding protein 14 22 kDa |
| 0.0196 | up | 1.8290 | PRO0628 | PRO0628 protein non-coding RNA |
| 0.0335 | up | 1.8288 | DUSP8 | dual specificity phosphatase 8 |
| 0.0179 | up | 1.8285 | RLF | rearranged L-myc fusion |
| 0.0492 | up | 1.8273 | HSF4 | heat shock transcription factor 4 transcript variant 1 |
| 0.0294 | up | 1.8261 | PDZD7 | PDZ domain containing 7 |
| 0.0159 | up | 1.8249 | RASSF1 | Ras association domain family member 1 transcript variant C |
| 0.0258 | up | 1.8237 | AKAP8 | A kinase anchor protein 8 |
| 0.0198 | up | 1.8224 | ING2 | inhibitor of growth family member 2 |
| 0.0025 | up | 1.8187 | FLNC | filamin C gamma |
| 0.0086 | up | 1.8090 | ING2 | inhibitor of growth family member 2 |
| 0.0403 | up | 1.8049 | LOC653086 | PREDICTED: similar to RAN-binding protein 2-like 1 isoform 2 transcript variant 6 |
| 0.0063 | up | 1.8044 | ALKBH5 | alkB alkylation repair homolog 5 |
| 0.0198 | up | 1.8043 | HCFC1R1 | host cell factor C1 regulator 1 transcript variant 3 |
| 0.0457 | up | 1.8031 | SFRS17A | splicing factor arginine/serine-rich 17A |
| 0.0026 | up | 1.8014 | RARA | retinoic acid receptor alpha transcript variant 1 |
| 0.0487 | up | 1.7978 | RYBP | RING1 and YY1 binding protein |
| 0.0034 | up | 1.7963 | TPI1 | triosephosphate isomerase 1 |
| 0.0211 | up | 1.7941 | PITPNC1 | phosphatidylinositol transfer protein cytoplasmic 1 transcript variant 2 |
| 0.0489 | up | 1.7922 | BTG1 | B-cell translocation gene 1 anti-proliferative |
| 0.0400 | up | 1.7912 | DHRS13 | dehydrogenase/reductase member 13 |
| 0.0008 | up | 1.7901 | LRRFIP1 | leucine rich repeat interacting protein 1 |
| 0.0183 | up | 1.7877 | TIPARP | TCDD-inducible poly polymerase |
| 0.0328 | up | 1.7869 | MLL5 | myeloid/lymphoid or mixed-lineage leukemia 5 transcript variant 2 |
| 0.0478 | up | 1.7835 | HERC3 | hect domain and RLD 3 |
| 0.0261 | up | 1.7826 | HM13 | histocompatibility 13 transcript variant 4 |
| 0.0168 | up | 1.7782 | CLK3 | CDC-like kinase 3 transcript variant phclk3 |
| 0.0131 | up | 1.7769 | SPRY1 | sprouty homolog 1 antagonist of FGF signaling transcript variant 1 |
| 0.0288 | up | 1.7766 | SNORD35A | small nucleolar RNA C/D box 35A non-coding RNA |
| 0.0053 | up | 1.7761 | ABL1 | c-abl oncogene 1 receptor tyrosine kinase transcript variant a |
| 0.0389 | up | 1.7752 | ARID3B | AT rich interactive domain 3B |
| 0.0101 | up | 1.7742 | SPN | sialophorin transcript variant 1 |
| 0.0013 | up | 1.7730 | XRCC2 | X-ray repair complementing defective repair in Chinese hamster cells 2 |
| 0.0017 | up | 1.7719 | HNRNPU | heterogeneous nuclear ribonucleoprotein U transcript variant 2 |
| 0.0048 | up | 1.7697 | BCL6 | B-cell CLL/lymphoma 6 transcript variant 1 |
| 0.0084 | up | 1.7687 | FLJ22184 | hypothetical protein FLJ22184 |
| 0.0291 | up | 1.7660 | CACNA2D3 | calcium channel voltage-dependent alpha 2/delta subunit 3 |

| | | | | |
|---|---|---|---|---|
| 0.0272 | up | 1.7653 | SNTA1 | syntrophin alpha 1 |
| 0.0234 | up | 1.7652 | DDB1 | PREDICTED: damage-specific DNA binding protein 1 127kDa transcript variant 4 |
| 0.0236 | up | 1.7630 | PHF21A | PHD finger protein 21A |
| 0.0029 | up | 1.7620 | UGP2 | UDP-glucose pyrophosphorylase 2 transcript variant 1 |
| 0.0263 | up | 1.7606 | LOC646123 | PREDICTED: hypothetical protein LOC646123 |
| 0.0065 | up | 1.7565 | PGAM4 | phosphoglycerate mutase family member 4 |
| 0.0339 | up | 1.7556 | FAM80B | family with sequence similarity 80 member B |
| 0.0198 | up | 1.7548 | LOC158301 | PREDICTED: hypothetical protein LOC158301 |
| 0.0423 | up | 1.7524 | MXI1 | MAX interactor 1 transcript variant 2 |
| 0.0156 | up | 1.7513 | SEMA3B | sema domain immunoglobulin domain short basic domain secreted 3B transcript variant 2 |
| 0.0468 | up | 1.7508 | AKAP12 | A kinase anchor protein 12 transcript variant 1 |
| 0.0195 | up | 1.7506 | RAB8B | RAB8B member RAS oncogene family |
| 0.0271 | up | 1.7472 | LOC440345 | PREDICTED: hypothetical protein LOC440345 transcript variant 6 |
| 0.0011 | up | 1.7452 | | xr14b10x1 NCI_CGAP_Lu28 cDNA clone IMAGE:2760091 3 sequence |
| 0.0022 | up | 1.7431 | GOLGA8B | golgi autoantigen golgin subfamily a 8B |
| 0.0391 | up | 1.7422 | PFKFB3 | 6-phosphofructo-2-kinase/fructose-26-biphosphatase 3 |
| 0.0210 | up | 1.7399 | LOC730525 | PREDICTED: hypothetical protein LOC730525 |
| 0.0027 | up | 1.7372 | JUND | jun D proto-oncogene |
| 0.0010 | up | 1.7329 | MYO3B | myosin IIIB |
| 0.0066 | up | 1.7329 | | BX108458 Soares_NhHMPu_S1 cDNA clone IMAGp998J154261 sequence |
| 0.0362 | up | 1.7321 | CTNND1 | catenin delta 1 transcript variant 1 |
| 0.0433 | up | 1.7289 | RBBP6 | retinoblastoma binding protein 6 transcript variant 3 |
| 0.0016 | up | 1.7288 | RNF126 | ring finger protein 126 |
| 0.0434 | up | 1.7277 | ANGPTL4 | angiopoietin-like 4 transcript variant 1 |
| 0.0417 | up | 1.7273 | SMOX | spermine oxidase transcript variant 4 |
| 0.0467 | up | 1.7272 | LONP1 | lon peptidase 1 mitochondrial nuclear gene encoding mitochondrial protein |
| 0.0016 | up | 1.7264 | ZNF483 | zinc finger protein 483 transcript variant 2 |
| 0.0488 | up | 1.7252 | LRRFIP2 | leucine rich repeat interacting protein 2 transcript variant 1 |
| 0.0123 | up | 1.7240 | VKORC1 | vitamin K epoxide reductase complex subunit 1 transcript variant 1 |
| 0.0039 | up | 1.7223 | PTGR2 | prostaglandin reductase 2 |
| 0.0073 | up | 1.7216 | LGALS8 | lectin galactoside-binding soluble 8 transcript variant 1 |
| 0.0270 | up | 1.7213 | PCSK5 | proprotein convertase subtilisin/kexin type 5 |
| 0.0430 | up | 1.7200 | C10orf67 | chromosome 10 open reading frame 67 |
| 0.0109 | up | 1.7199 | ZNF503 | zinc finger protein 503 |
| 0.0104 | up | 1.7183 | CTAGE6 | CTAGE family member 6 |
| 0.0045 | up | 1.7177 | CDKN2D | cyclin-dependent kinase inhibitor 2D transcript variant 2 |
| 0.0220 | up | 1.7164 | UCHL1 | ubiquitin carboxyl-terminal esterase L1 |
| 0.0083 | up | 1.7153 | PHF13 | PHD finger protein 13 |
| 0.0086 | up | 1.7144 | PIAS2 | protein inhibitor of activated STAT 2 transcript variant beta |
| 0.0198 | up | 1.7131 | LOC653125 | PREDICTED: similar to Golgi autoantigen golgin subfamily A member 2 transcript variant 2 |
| 0.0371 | up | 1.7116 | CD109 | CD109 molecule |
| 0.0094 | up | 1.7088 | P4HA1 | procollagen-proline 2-oxoglutarate 4-dioxygenase alpha polypeptide I transcript variant 2 |
| 0.0270 | up | 1.7073 | PHKG1 | phosphorylase kinase gamma 1 |
| 0.0199 | up | 1.7043 | LZTS2 | leucine zipper putative tumor suppressor 2 |
| 0.0074 | up | 1.7015 | SAP30 | Sin3A-associated protein 30kDa |
| 0.0145 | up | 1.7001 | PPM1B | protein phosphatase 1B magnesium-dependent beta isoform transcript variant 4 |
| 0.0015 | up | 1.6998 | MBTD1 | mbt domain containing 1 |

| | | | | |
|---|---|---|---|---|
| 0.0176 | up | 1.6993 | LOC401152 | HCV F-transactivated protein 1 |
| 0.0211 | up | 1.6974 | RAPGEFL1 | Rap guanine nucleotide exchange factor -like 1 |
| 0.0126 | up | 1.6966 | HK1 | hexokinase 1 nuclear gene encoding mitochondrial protein transcript variant 5 |
| 0.0032 | up | 1.6963 | TMEM189-UBE2V1 | TMEM189-UBE2V1 transcript variant 1 |
| 0.0364 | up | 1.6962 | ARID5A | AT rich interactive domain 5A |
| 0.0477 | up | 1.6950 | GRPEL1 | GrpE-like 1 mitochondrial nuclear gene encoding mitochondrial protein |
| 0.0113 | up | 1.6936 | ANKRD47 | ankyrin repeat domain 47 |
| 0.0367 | up | 1.6935 | PPFIBP1 | PTPRF interacting protein binding protein 1 transcript variant 1 |
| 0.0305 | up | 1.6910 | SEMA6D | sema domain transmembrane domain and cytoplasmic domain 6D transcript variant 5 |
| 0.0007 | up | 1.6907 | | xj89b12x1 Soares_NFL_T_GBC_S1 cDNA clone IMAGE:2664383 3 sequence |
| 0.0253 | up | 1.6888 | GOLGA8B | golgi autoantigen golgin subfamily a 8B |
| 0.0319 | up | 1.6883 | INADL | InaD-like |
| 0.0067 | up | 1.6880 | ARHGEF5 | Rho guanine nucleotide exchange factor 5 |
| 0.0485 | up | 1.6874 | | AGENCOURT_6796899 NIH_MGC_85 cDNA clone IMAGE:5787825 5 sequence |
| 0.0009 | up | 1.6834 | | MR1-GN0172-061100-005-h03 GN0172 cDNA sequence |
| 0.0108 | up | 1.6799 | DST | dystonin transcript variant 1 |
| 0.0008 | up | 1.6798 | FAM115A | family with sequence similarity 115 member A |
| 0.0118 | up | 1.6784 | RASSF6 | Ras association domain family member 6 transcript variant 1 |
| 0.0169 | up | 1.6777 | SEMA3B | sema domain immunoglobulin domain short basic domain secreted 3B transcript variant 2 |
| 0.0168 | up | 1.6764 | FOXO3 | forkhead box O3 transcript variant 2 |
| 0.0114 | up | 1.6757 | NFAT5 | nuclear factor of activated T-cells 5 tonicity-responsive transcript variant 5 |
| 0.0001 | up | 1.6741 | SEMA3E | sema domain immunoglobulin domain short basic domain secreted 3E |
| 0.0166 | up | 1.6740 | SPRY1 | sprouty homolog 1 antagonist of FGF signaling transcript variant 2 |
| 0.0024 | up | 1.6732 | C14orf82 | PREDICTED: chromosome 14 open reading frame 82 |
| 0.0031 | up | 1.6698 | | ny62g05s1 NCI_CGAP_GCB1 cDNA clone IMAGE:1282904 3 sequence |
| 0.0142 | up | 1.6668 | LGALS8 | lectin galactoside-binding soluble 8 transcript variant 4 |
| 0.0387 | up | 1.6664 | PDXP | pyridoxal phosphatase |
| 0.0140 | up | 1.6654 | P4HB | procollagen-proline 2-oxoglutarate 4-dioxygenase beta polypeptide |
| 0.0486 | up | 1.6650 | LOC644782 | PREDICTED: similar to implantation-associated protein |
| 0.0218 | up | 1.6649 | ARRDC3 | arrestin domain containing 3 |
| 0.0076 | up | 1.6646 | LOC400027 | PREDICTED: hypothetical gene supported by BC047417 transcript variant 2 |
| 0.0046 | up | 1.6645 | TUBA4A | tubulin alpha 4a |
| 0.0353 | up | 1.6637 | SH2D5 | PREDICTED: SH2 domain containing 5 |
| 0.0469 | up | 1.6630 | | cDNA FLJ14199 fis clone NT2RP3002713 |
| 0.0005 | up | 1.6630 | ZNF394 | zinc finger protein 394 |
| 0.0413 | up | 1.6629 | SLC6A10P | solute carrier family 6 member 10 on chromosome 16 |
| 0.0245 | up | 1.6609 | VPS37D | vacuolar protein sorting 37 homolog D |
| 0.0336 | up | 1.6588 | FLJ10213 | hypothetical protein FLJ10213 |
| 0.0041 | up | 1.6584 | TRIO | triple functional domain |
| 0.0014 | up | 1.6577 | CCBE1 | collagen and calcium binding EGF domains 1 |
| 0.0016 | up | 1.6559 | SLC35E1 | solute carrier family 35 member E1 |
| 0.0308 | up | 1.6513 | ZNF483 | zinc finger protein 483 transcript variant 2 |
| 0.0048 | up | 1.6508 | CATSPER2 | cation channel sperm associated 2 transcript variant 4 |
| 0.0049 | up | 1.6506 | WDR74 | WD repeat domain 74 |
| 0.0263 | up | 1.6500 | MXI1 | MAX interactor 1 transcript variant 1 |
| 0.0410 | up | 1.6498 | DDX41 | DEAD box polypeptide 41 |

| | | | | |
|---|---|---|---|---|
| 0.0019 | up | 1.6489 | TMEM17 | transmembrane protein 17 |
| 0.0021 | up | 1.6478 | CHRNA5 | cholinergic receptor nicotinic alpha 5 |
| 0.0081 | up | 1.6477 | ZNF600 | zinc finger protein 600 |
| 0.0452 | up | 1.6475 | CORO6 | coronin 6 |
| 0.0057 | up | 1.6433 | SPTBN1 | spectrin beta non-erythrocytic 1  transcript variant 1 |
| 0.0087 | up | 1.6417 | CARHSP1 | calcium regulated heat stable protein 1 24kDa  transcript variant 2 |
| 0.0049 | up | 1.6411 | RNASE4 | ribonuclease RNase A family 4  transcript variant 1 |
| 0.0037 | up | 1.6409 | PCDHB19P | protocadherin beta 19 pseudogene  non-coding RNA |
| 0.0022 | up | 1.6408 | SMS | spermine synthase |
| 0.0392 | up | 1.6394 | HIST2H2AB | histone cluster 2 H2ab |
| 0.0034 | up | 1.6392 | KRT80 | keratin 80  transcript variant 1 |
| 0.0074 | up | 1.6383 | NUBPL | nucleotide binding protein-like |
| 0.0008 | up | 1.6382 | NPIP | nuclear pore complex interacting protein |
| 0.0311 | up | 1.6357 | MACF1 | microtubule-actin crosslinking factor 1  transcript variant 1 |
| 0.0012 | up | 1.6354 | CYCSL1 | cytochrome c somatic-like 1  on chromosome 6 |
| 0.0186 | up | 1.6351 | PTP4A2 | PREDICTED:  protein tyrosine phosphatase type IVA member 2 transcript variant 5 |
| 0.0081 | up | 1.6349 | | zr87e09r1 NCI_CGAP_GCB1  cDNA clone IMAGE:682696 5  sequence |
| 0.0050 | up | 1.6346 | RNASE4 | ribonuclease RNase A family 4  transcript variant 1 |
| 0.0409 | up | 1.6339 | WTAP | Wilms tumor 1 associated protein  transcript variant 1 |
| 0.0323 | up | 1.6329 | | ta96c03x1 NCI_CGAP_Lu26  cDNA clone IMAGE:2051908 3  sequence |
| 0.0478 | up | 1.6326 | CHRNB1 | cholinergic receptor nicotinic beta 1 |
| 0.0009 | up | 1.6325 | SMS | spermine synthase |
| 0.0235 | up | 1.6323 | MYH9 | myosin heavy chain 9 non-muscle |
| 0.0002 | up | 1.6320 | YRDC | yrdC domain containing |
| 0.0200 | up | 1.6297 | HM13 | histocompatibility  13  transcript variant 4 |
| 0.0192 | up | 1.6296 | IGFBP6 | insulin-like growth factor binding protein 6 |
| 0.0026 | up | 1.6288 | HSPC268 | hypothetical protein HSPC268 |
| 0.0021 | up | 1.6286 | C6orf170 | chromosome 6 open reading frame 170 |
| 0.0073 | up | 1.6282 | LOC648852 | PREDICTED:  hypothetical protein LOC648852 |
| 0.0095 | up | 1.6277 | LOC642678 | PREDICTED:  similar to myeloid/lymphoid or mixed-lineage leukemia 3 isoform 2 |
| 0.0318 | up | 1.6262 | EIF5 | eukaryotic translation initiation factor 5  transcript variant 2 |
| 0.0047 | up | 1.6234 | SLC36A4 | solute carrier family 36  member 4 |
| 0.0034 | up | 1.6233 | EPS8L1 | EPS8-like 1  transcript variant 1 |
| 0.0275 | up | 1.6217 | FLJ43692 | ARHGEF5-like |
| 0.0031 | up | 1.6209 | RNUXA | RNA U small nuclear RNA export adaptor |
| 0.0052 | up | 1.6189 | FAM63A | family with sequence similarity 63 member A  transcript variant 1 |
| 0.0278 | up | 1.6174 | ANG | angiogenin ribonuclease RNase A family 5  transcript variant 2 |
| 0.0361 | up | 1.6172 | MFSD10 | major facilitator superfamily domain containing 10 |
| 0.0334 | up | 1.6169 | UBC | ubiquitin C |
| 0.0210 | up | 1.6167 | RAB6IP1 | RAB6 interacting protein 1 |
| 0.0012 | up | 1.6159 | | tq42f05x1 NCI_CGAP_Ut1  cDNA clone IMAGE:2211489 3  sequence |
| 0.0002 | up | 1.6142 | PFKL | phosphofructokinase liver  transcript variant 2 |
| 0.0028 | up | 1.6141 | PIP5K2B | phosphatidylinositol-4-phosphate 5-kinase type II beta  transcript variant 2 |
| 0.0082 | up | 1.6123 | ANKRD44 | ankyrin repeat domain 44 |
| 0.0332 | up | 1.6120 | LOC493754 | PREDICTED:  hypothetical LOC493754 |
| 0.0154 | up | 1.6108 | RASSF7 | Ras association  domain family  member 7 |
| 0.0025 | up | 1.6107 | | te46f04x1 Soares_NhHMPu_S1  cDNA clone IMAGE:2089759 3  sequence |

| | | | | |
|---|---|---|---|---|
| 0.0029 | up | 1.6101 | PRKRIP1 | PRKR interacting protein 1 |
| 0.0381 | up | 1.6098 | KIAA2010 | KIAA2010 transcript variant 2 |
| 0.0058 | up | 1.6091 | PGRMC2 | progesterone receptor membrane component 2 |
| 0.0016 | up | 1.6077 | ANG | angiogenin ribonuclease RNase A family 5 transcript variant 2 |
| 0.0026 | up | 1.6049 | GRIPAP1 | GRIP1 associated protein 1 transcript variant 2 |
| 0.0443 | up | 1.6042 | PVR | poliovirus receptor |
| 0.0472 | up | 1.6032 | LOC653663 | PREDICTED: similar to anaphase promoting complex subunit 1 transcript variant 2 |
| 0.0016 | up | 1.6030 | BLZF1 | basic leucine zipper nuclear factor 1 |
| 0.0389 | up | 1.6029 | LOC653319 | hypothetical protein LOC653319 |
| 0.0181 | up | 1.6024 | TMSB10 | thymosin beta 10 |
| 0.0203 | up | 1.6023 | CLK3 | CDC-like kinase 3 transcript variant phclk3 |
| 0.0052 | up | 1.6017 | GDPD1 | glycerophosphodiester phosphodiesterase domain containing 1 |
| 0.0075 | up | 1.5996 | MAPK8IP3 | mitogen-activated protein kinase 8 interacting protein 3 transcript variant 2 |
| 0.0080 | up | 1.5994 | TPPP3 | tubulin polymerization-promoting protein family member 3 |
| 0.0393 | up | 1.5993 | NR2C2 | nuclear receptor subfamily 2 group C member 2 |
| 0.0374 | up | 1.5992 | HCG4 | HLA complex group 4 non-coding RNA |
| 0.0450 | up | 1.5977 | PHF1 | PHD finger protein 1 transcript variant 2 |
| 0.0137 | up | 1.5969 | ProSAPiP1 | ProSAPiP1 protein |
| 0.0211 | up | 1.5967 | OR2A9P | olfactory receptor family 2 subfamily A member 9 pseudogene non-coding RNA |
| 0.0059 | up | 1.5960 | RHBDL2 | rhomboid veinlet-like 2 |
| 0.0024 | up | 1.5923 | TDRD1 | tudor domain containing 1 |
| 0.0325 | up | 1.5909 | ARID5A | AT rich interactive domain 5A |
| 0.0002 | up | 1.5903 | SHROOM4 | shroom family member 4 |
| 0.0186 | up | 1.5868 | CCNY | cyclin Y transcript variant 1 |
| 0.0092 | up | 1.5867 | LOC644852 | PREDICTED: hypothetical protein LOC644852 transcript variant 2 |
| 0.0442 | up | 1.5864 | WHDC1L1 | WAS protein homology region 2 domain containing 1-like 1 non-coding RNA |
| 0.0432 | up | 1.5846 | DEDD2 | death effector domain containing 2 |
| 0.0424 | up | 1.5832 | RGPD1 | RANBP2-like and GRIP domain containing 1 |
| 0.0014 | up | 1.5831 | MAPK7 | mitogen-activated protein kinase 7 transcript variant 2 |
| 0.0415 | up | 1.5827 | STRC | stereocilin |
| 0.0263 | up | 1.5821 | EIF4H | eukaryotic translation initiation factor 4H transcript variant 2 |
| 0.0009 | up | 1.5801 | GALNT3 | UDP-N-acetyl-alpha-D-galactosamine:polypeptide N-acetylgalactosaminyltransferase 3 |
| 0.0160 | up | 1.5786 | CTAGE6 | PREDICTED: CTAGE family member 6 |
| 0.0298 | up | 1.5780 | CRKL | v-crk sarcoma virus CT10 oncogene homolog -like |
| 0.0178 | up | 1.5775 | DST | dystonin transcript variant 1e |
| 0.0482 | up | 1.5772 | RBPJ | recombination signal binding protein for immunoglobulin kappa J region transcript variant 4 |
| 0.0393 | up | 1.5771 | PDK1 | pyruvate dehydrogenase kinase isozyme 1 nuclear gene encoding mitochondrial protein |
| 0.0008 | up | 1.5759 | C3orf34 | chromosome 3 open reading frame 34 |
| 0.0415 | up | 1.5754 | ABCA1 | ATP-binding cassette sub-family A member 1 |
| 0.0044 | up | 1.5745 | LOC646786 | PREDICTED: similar to Afadin |
| 0.0017 | up | 1.5742 | LOC399900 | hypothetical gene supported by AK093779 |
| 0.0158 | up | 1.5739 | PPL | periplakin |
| 0.0202 | up | 1.5732 | UBE2H | ubiquitin-conjugating enzyme E2H transcript variant 1 |
| 0.0178 | up | 1.5729 | KIAA1267 | KIAA1267 |
| 0.0331 | up | 1.5707 | | PREDICTED: hypothetical LOC388727 |
| 0.0163 | up | 1.5706 | RNF126 | ring finger protein 126 |
| 0.0108 | up | 1.5705 | TPSAB1 | tryptase alpha/beta 1 |

| | | | | |
|---|---|---|---|---|
| 0.0470 | up | 1.5699 | USP3 | ubiquitin specific peptidase 3 |
| 0.0010 | up | 1.5699 | S100A14 | S100 calcium binding protein A14 |
| 0.0037 | up | 1.5694 | SPDYA | speedy homolog A transcript variant 2 |
| 0.0145 | up | 1.5692 | CIC | capicua homolog |
| 0.0194 | up | 1.5676 | ZNF432 | zinc finger protein 432 |
| 0.0263 | up | 1.5675 | SGK269 | NKF3 kinase family member |
| 0.0195 | up | 1.5672 | C9orf30 | chromosome 9 open reading frame 30 |
| 0.0100 | up | 1.5670 | LOC647389 | PREDICTED: hypothetical protein LOC647389 |
| 0.0483 | up | 1.5668 | PKM2 | pyruvate kinase muscle transcript variant 3 |
| 0.0439 | up | 1.5665 | LOC645349 | PREDICTED: hypothetical protein LOC645349 |
| 0.0010 | up | 1.5647 | NLRP8 | NLR family pyrin domain containing 8 |
| 0.0389 | up | 1.5638 | HS6ST2 | heparan sulfate 6-O-sulfotransferase 2 transcript variant L |
| 0.0486 | up | 1.5626 | TBC1D8 | TBC1 domain family member 8 |
| 0.0016 | up | 1.5622 | ZNF682 | zinc finger protein 682 transcript variant 1 |
| 0.0398 | up | 1.5611 | ZNF821 | zinc finger protein 821 |
| 0.0246 | up | 1.5600 | MPZL3 | myelin protein zero-like 3 |
| 0.0087 | up | 1.5599 | | hypothetical protein LOC338620 partial cds |
| 0.0238 | up | 1.5596 | C12orf24 | chromosome 12 open reading frame 24 |
| 0.0004 | up | 1.5590 | C1orf176 | chromosome 1 open reading frame 176 |
| 0.0086 | up | 1.5581 | ZNF786 | zinc finger protein 786 |
| 0.0052 | up | 1.5566 | SSTR2 | somatostatin receptor 2 |
| 0.0041 | up | 1.5559 | LMOD3 | leiomodin 3 |
| 0.0308 | up | 1.5558 | C6orf106 | chromosome 6 open reading frame 106 transcript variant 1 |
| 0.0084 | up | 1.5549 | ZNF69 | zinc finger protein 69 |
| 0.0305 | up | 1.5546 | ING1 | inhibitor of growth family member 1 transcript variant 1 |
| 0.0429 | up | 1.5520 | DHX57 | DEAH box polypeptide 57 transcript variant 3 |
| 0.0092 | up | 1.5514 | USP49 | ubiquitin specific peptidase 49 |
| 0.0493 | up | 1.5512 | LOC391045 | PREDICTED: similar to Solute carrier family 2 facilitated glucose transporter member 3 |
| 0.0292 | up | 1.5500 | PDZD8 | PDZ domain containing 8 |
| 0.0368 | up | 1.5488 | MAP4K5 | mitogen-activated protein kinase kinase kinase kinase 5 transcript variant 2 |
| 0.0015 | up | 1.5484 | DDX51 | DEAD box polypeptide 51 |
| 0.0015 | up | 1.5478 | FUT6 | fucosyltransferase 6 fucosyltransferase) transcript variant 1 |
| 0.0068 | up | 1.5476 | UGP2 | UDP-glucose pyrophosphorylase 2 transcript variant 1 |
| 0.0033 | up | 1.5468 | SOX4 | SRY -box 4 |
| 0.0294 | up | 1.5468 | CRYBB2 | crystallin beta B2 |
| 0.0390 | up | 1.5455 | LOC151579 | PREDICTED: similar to basic leucine zipper and W2 domains 1 |
| 0.0052 | up | 1.5452 | FLJ44124 | hypothetical protein LOC641737 |
| 0.0012 | up | 1.5449 | ZNF14 | zinc finger protein 14 |
| 0.0013 | up | 1.5446 | C14orf153 | chromosome 14 open reading frame 153 |
| 0.0096 | up | 1.5440 | NEU4 | sialidase 4 |
| 0.0013 | up | 1.5439 | DUSP19 | dual specificity phosphatase 19 |
| 0.0002 | up | 1.5432 | GSTTP2 | glutathione S-transferase theta pseudogene 2 non-coding RNA XM_941198 XM_945014 XM_945016 |
| 0.0052 | up | 1.5426 | ERF | Ets2 repressor factor |
| 0.0135 | up | 1.5420 | ADAM9 | ADAM metallopeptidase domain 9 transcript variant 1 |
| 0.0065 | up | 1.5413 | HSPB7 | heat shock 27kDa protein family member 7 |
| 0.0039 | up | 1.5389 | ZNF652 | zinc finger protein 652 |
| 0.0325 | up | 1.5387 | LOC440349 | PREDICTED: similar to nuclear pore complex interacting protein transcript variant 1 |

| | | | | |
|---|---|---|---|---|
| 0.0003 | up | 1.5381 | | wi12b07x1 NCI_CGAP_Co16 cDNA clone IMAGE:2390005 3 sequence |
| 0.0452 | up | 1.5370 | CDH5 | cadherin 5 type 2 VE-cadherin |
| 0.0173 | up | 1.5351 | SEC61G | Sec61 gamma subunit transcript variant 2 |
| 0.0140 | up | 1.5346 | ZNF654 | zinc finger protein 654 |
| 0.0046 | up | 1.5330 | KCNK6 | potassium channel subfamily K member 6 |
| 0.0102 | up | 1.5325 | FAM73A | family with sequence similarity 73 member A |
| 0.0264 | up | 1.5317 | TncRNA | PREDICTED: trophoblast-derived noncoding RNA misc RNA |
| 0.0189 | up | 1.5314 | GOLGA6B | golgi autoantigen golgin subfamily a 6B |
| 0.0002 | up | 1.5306 | FAM119A | family with sequence similarity 119 member A |
| 0.0010 | up | 1.5293 | DHX32 | DEAH box polypeptide 32 |
| 0.0292 | up | 1.5283 | PTPRM | protein tyrosine phosphatase receptor type M |
| 0.0423 | up | 1.5281 | MAP4 | microtubule-associated protein 4 transcript variant 3 |
| 0.0337 | up | 1.5279 | LOC649270 | PREDICTED: similar to aminopeptidase puromycin sensitive transcript variant 12 |
| 0.0374 | up | 1.5271 | DRD3 | dopamine receptor D3 transcript variant a |
| 0.0153 | up | 1.5268 | RASD2 | RASD family member 2 |
| 0.0051 | up | 1.5260 | FAM38A | family with sequence similarity 38 member A |
| 0.0197 | up | 1.5259 | SVIL | supervillin transcript variant 1 |
| 0.0137 | up | 1.5258 | EIF4H | eukaryotic translation initiation factor 4H transcript variant 1 |
| 0.0295 | up | 1.5246 | WBP5 | WW domain binding protein 5 transcript variant 2 |
| 0.0363 | up | 1.5241 | | partial ; ID YG39-1C |
| 0.0116 | up | 1.5241 | LOC653829 | PREDICTED: similar to Williams Beuren syndrome chromosome region 19 transcript variant 6 |
| 0.0059 | up | 1.5232 | LOC648059 | PREDICTED: hypothetical protein LOC648059 |
| 0.0242 | up | 1.5227 | MTMR3 | myotubularin related protein 3 transcript variant 3 |
| 0.0186 | up | 1.5223 | PTP4A2 | PREDICTED: protein tyrosine phosphatase type IVA member 2 transcript variant 9 |
| 0.0124 | up | 1.5223 | | wf40a08x1 Soares_NFL_T_GBC_S1 cDNA clone IMAGE:2358038 3 similar to contains Alu repetitive element; sequence |
| 0.0314 | up | 1.5221 | | AV645572 GLC cDNA clone GLCABG03 3 sequence |
| 0.0150 | up | 1.5219 | GPRC5A | G protein-coupled receptor family C group 5 member A |
| 0.0403 | up | 1.5219 | PER1 | period homolog 1 |
| 0.0027 | up | 1.5214 | JARID1B | jumonji AT rich interactive domain 1B |
| 0.0158 | up | 1.5210 | LOC643882 | PREDICTED: hypothetical protein LOC643882 |
| 0.0466 | up | 1.5210 | LATS1 | LATS large tumor suppressor homolog 1 |
| 0.0024 | up | 1.5208 | LOC645558 | PREDICTED: hypothetical LOC645558 |
| 0.0401 | up | 1.5203 | DKFZp761E198 | DKFZp761E198 protein |
| 0.0350 | up | 1.5194 | SEC61G | Sec61 gamma subunit transcript variant 1 |
| 0.0482 | up | 1.5192 | SEC24B | SEC24 family member B transcript variant 1 |
| 0.0450 | up | 1.5189 | | DA831045 PLACE1 cDNA clone PLACE1006268 5 sequence |
| 0.0015 | up | 1.5183 | MBD4 | methyl-CpG binding domain protein 4 |
| 0.0129 | up | 1.5179 | LOC730316 | PREDICTED: similar to Nuclear envelope pore membrane protein POM 121 |
| 0.0179 | up | 1.5178 | WDR26 | WD repeat domain 26 |
| 0.0003 | up | 1.5173 | TDRD1 | tudor domain containing 1 |
| 0.0363 | up | 1.5162 | C9orf130 | PREDICTED: chromosome 9 open reading frame 130 |
| 0.0077 | up | 1.5146 | BCKDHA | branched chain keto acid dehydrogenase E1 alpha polypeptide |
| 0.0190 | up | 1.5145 | PAQR6 | progestin and adipoQ receptor family member VI transcript variant 1 |
| 0.0007 | up | 1.5144 | ZNF549 | zinc finger protein 549 |
| 0.0478 | up | 1.5138 | FRS2 | fibroblast growth factor receptor substrate 2 transcript variant 1 |
| 0.0032 | up | 1.5134 | GPR1 | G protein-coupled receptor 1 |
| 0.0043 | up | 1.5130 | ALPP | alkaline phosphatase placental |

| | | | | |
|---|---|---|---|---|
| 0.0244 | up | 1.5117 | RP11-529I10.4 | deleted in a mouse model of primary ciliary dyskinesia |
| 0.0009 | up | 1.5112 | FBLN1 | fibulin 1 transcript variant C |
| 0.0103 | up | 1.5093 | LOC730347 | PREDICTED: similar to rho guanine nucleotide exchange factor 5 |
| 0.0388 | up | 1.5091 | PDE4B | phosphodiesterase 4B cAMP-specific transcript variant a |
| 0.0201 | up | 1.5091 | PRSS8 | protease serine 8 |
| 0.0440 | up | 1.5091 | HPS4 | Hermansky-Pudlak syndrome 4 transcript variant 2 |
| 0.0012 | up | 1.5090 | LOC642946 | PREDICTED: hypothetical protein LOC642945 transcript variant 1 |
| 0.0106 | up | 1.5089 | AFAP1L1 | actin filament associated protein 1-like 1 |
| 0.0420 | up | 1.5088 | SLC29A4 | solute carrier family 29 member 4 |
| 0.0052 | up | 1.5087 | | UI-CF-EN1-acz-c-09-0-UIs1 UI-CF-EN1 cDNA clone UI-CF-EN1-acz-c-09-0-UI 3 sequence |
| 0.0200 | up | 1.5086 | RELA | v-rel reticuloendotheliosis viral oncogene homolog A |
| 0.0084 | up | 1.5083 | PCDH17 | protocadherin 17 |
| 0.0094 | up | 1.5082 | BAGE5 | B melanoma antigen family member 5 |
| 0.0184 | up | 1.5077 | USP42 | ubiquitin specific peptidase 42 |
| 0.0027 | up | 1.5077 | MLL3 | myeloid/lymphoid or mixed-lineage leukemia 3 |
| 0.0483 | up | 1.5077 | RAB12 | RAB12 member RAS oncogene family |
| 0.0377 | up | 1.5061 | FGD4 | FYVE RhoGEF and PH domain containing 4 |
| 0.0435 | up | 1.5055 | TBC1D23 | TBC1 domain family member 23 |
| 0.0039 | up | 1.5039 | | UI-H-BI2-agn-g-04-0-UIs1 NCI_CGAP_Sub4 cDNA clone IMAGE:2725039 3 sequence |
| 0.0033 | up | 1.5037 | SELO | selenoprotein O |
| 0.0190 | up | 1.5036 | NKTR | natural killer-tumor recognition sequence |
| 0.0316 | up | 1.5035 | PLXNA3 | plexin A3 |
| 0.0211 | up | 1.5031 | SAPS2 | PREDICTED: SAPS domain family member 2 transcript variant 2 |
| 0.0057 | up | 1.5030 | PRO1853 | hypothetical protein PRO1853 transcript variant 1 |
| 0.0020 | up | 1.5028 | HCG2P7 | HLA complex group 2 pseudogene 7 non-coding RNA |
| 0.0022 | up | 1.5027 | C7orf54 | chromosome 7 open reading frame 54 |
| 0.0054 | up | 1.5026 | CHD1L | chromodomain helicase DNA binding protein 1-like |
| 0.0023 | up | 1.5026 | FLJ46309 | hypothetical protein LOC649598 |
| 0.0478 | up | 1.5024 | RFPL3S | RFPL3 antisense RNA non-coding RNA |
| 0.0085 | up | 1.5023 | NXNL1 | nucleoredoxin-like 1 |
| 0.0335 | up | 1.5018 | BRD4 | bromodomain containing 4 transcript variant long |
| 0.0012 | up | 1.5018 | C6orf206 | chromosome 6 open reading frame 206 |
| 0.0494 | up | 1.5017 | SLC25A36 | solute carrier family 25 member 36 |
| 0.0018 | up | 1.5017 | SRRM2 | serine/arginine repetitive matrix 2 |
| 0.0303 | up | 1.5013 | LOC400890 | PREDICTED: hypothetical LOC400890 |
| 0.0408 | up | 1.5009 | TES | testis derived transcript transcript variant 1 |
| 0.0194 | down | -1.5000 | PRKAR1A | protein kinase cAMP-dependent regulatory type I alpha transcript variant 1 |
| 0.0003 | down | -1.5003 | RFC4 | replication factor C 4 37kDa transcript variant 2 |
| 0.0444 | down | -1.5006 | LOC642197 | PREDICTED: similar to Protein FAM82B |
| 0.0355 | down | -1.5008 | NKIRAS1 | NFKB inhibitor interacting Ras-like 1 |
| 0.0169 | down | -1.5008 | TMEM141 | transmembrane protein 141 |
| 0.0013 | down | -1.5014 | BAT5 | HLA-B associated transcript 5 |
| 0.0255 | down | -1.5016 | NGFRAP1 | nerve growth factor receptor associated protein 1 transcript variant 1 |
| 0.0175 | down | -1.5017 | GBA | glucosidase beta; acid transcript variant 3 |
| 0.0023 | down | -1.5018 | NDUFAB1 | NADH dehydrogenase 1 alpha/beta subcomplex 1 8kDa |
| 0.0123 | down | -1.5022 | AHCYL2 | S-adenosylhomocysteine hydrolase-like 2 |
| 0.0036 | down | -1.5024 | MLH1 | mutL homolog 1 colon cancer nonpolyposis type 2 |

| | | | | |
|---|---|---|---|---|
| 0.0114 | down | -1.5032 | ADO | 2-aminoethanethiol dioxygenase |
| 0.0253 | down | -1.5033 | BCDIN3D | BCDIN3 domain containing |
| 0.0192 | down | -1.5035 | CYB5R1 | cytochrome b5 reductase 1 |
| 0.0263 | down | -1.5037 | TOR1B | torsin family 1 member B |
| 0.0217 | down | -1.5039 | UBE2A | ubiquitin-conjugating enzyme E2A transcript variant 2 |
| 0.0102 | down | -1.5042 | PPAP2B | phosphatidic acid phosphatase type 2B transcript variant 2 |
| 0.0320 | down | -1.5045 | ZNF480 | zinc finger protein 480 |
| 0.0090 | down | -1.5052 | ABHD3 | abhydrolase domain containing 3 |
| 0.0181 | down | -1.5052 | TXNDC9 | thioredoxin domain containing 9 |
| 0.0063 | down | -1.5061 | ITFG2 | integrin alpha FG-GAP repeat containing 2 |
| 0.0187 | down | -1.5072 | FLAD1 | FAD1 flavin adenine dinucleotide synthetase homolog transcript variant 2 |
| 0.0081 | down | -1.5076 | C8orf33 | chromosome 8 open reading frame 33 |
| 0.0429 | down | -1.5085 | C20orf177 | chromosome 20 open reading frame 177 |
| 0.0003 | down | -1.5085 | HSDL2 | hydroxysteroid dehydrogenase like 2 |
| 0.0093 | down | -1.5086 | TSSC1 | tumor suppressing subtransferable candidate 1 |
| 0.0326 | down | -1.5095 | SPRYD4 | SPRY domain containing 4 |
| 0.0373 | down | -1.5096 | XPO4 | exportin 4 |
| 0.0382 | down | -1.5099 | TMEM30A | transmembrane protein 30A |
| 0.0345 | down | -1.5100 | MTMR10 | myotubularin related protein 10 |
| 0.0043 | down | -1.5105 | PARP12 | poly polymerase family member 12 |
| 0.0229 | down | -1.5107 | C3orf1 | chromosome 3 open reading frame 1 |
| 0.0177 | down | -1.5110 | WDR77 | WD repeat domain 77 |
| 0.0196 | down | -1.5117 | TRAPPC2 | trafficking protein particle complex 2 transcript variant 2 |
| 0.0267 | down | -1.5119 | EFHA2 | EF-hand domain family member A2 |
| 0.0221 | down | -1.5120 | CASP6 | caspase 6 apoptosis-related cysteine peptidase transcript variant alpha |
| 0.0050 | down | -1.5121 | FIG4 | FIG4 homolog |
| 0.0140 | down | -1.5123 | UNG | uracil-DNA glycosylase transcript variant 1 |
| 0.0419 | down | -1.5133 | CSTF3 | cleavage stimulation factor 3' pre-RNA subunit 3 77kDa transcript variant 2 |
| 0.0028 | down | -1.5137 | SNX17 | sorting nexin 17 |
| 0.0116 | down | -1.5138 | MRPS12 | mitochondrial ribosomal protein S12 nuclear gene encoding mitochondrial protein transcript variant 3 |
| 0.0127 | down | -1.5139 | CCT7 | chaperonin containing TCP1 subunit 7 transcript variant 1 |
| 0.0116 | down | -1.5140 | CIAPIN1 | cytokine induced apoptosis inhibitor 1 |
| 0.0170 | down | -1.5148 | C12orf4 | chromosome 12 open reading frame 4 |
| 0.0390 | down | -1.5157 | TRMT12 | tRNA methyltransferase 12 homolog |
| 0.0183 | down | -1.5166 | MOCS2 | molybdenum cofactor synthesis 2 transcript variant 1 |
| 0.0213 | down | -1.5167 | PSMG1 | proteasome assembly chaperone 1 transcript variant 2 |
| 0.0384 | down | -1.5176 | C14orf129 | chromosome 14 open reading frame 129 |
| 0.0219 | down | -1.5189 | LOC153364 | similar to metallo-beta-lactamase superfamily protein |
| 0.0246 | down | -1.5193 | GLRX2 | glutaredoxin 2 transcript variant 2 |
| 0.0287 | down | -1.5194 | GSTZ1 | glutathione transferase zeta 1 transcript variant 2 |
| 0.0268 | down | -1.5197 | BRD8 | bromodomain containing 8 transcript variant 3 |
| 0.0175 | down | -1.5199 | C1orf122 | chromosome 1 open reading frame 122 |
| 0.0168 | down | -1.5203 | LOC653226 | PREDICTED: similar to Signal recognition particle 9 kDa protein |
| 0.0433 | down | -1.5209 | MUT | methylmalonyl Coenzyme A mutase nuclear gene encoding mitochondrial protein |
| 0.0001 | down | -1.5216 | Magmas | mitochondria-associated protein involved in granulocyte-macrophage colony-stimulating factor signal transduction nuclear gene encoding mitochondrial protein |
| 0.0430 | down | -1.5217 | USP21 | ubiquitin specific peptidase 21 transcript variant 1 |
| 0.0049 | down | -1.5218 | PYCR2 | pyrroline-5-carboxylate reductase family member 2 |

| | | | | |
|---|---|---|---|---|
| 0.0035 | down | -1.5219 | MDH1 | malate dehydrogenase 1 NAD |
| 0.0327 | down | -1.5219 | MPV17 | MpV17 mitochondrial inner membrane protein  nuclear gene encoding mitochondrial protein |
| 0.0294 | down | -1.5220 | SNX19 | sorting nexin 19 |
| 0.0329 | down | -1.5220 | TMEM138 | transmembrane protein 138 |
| 0.0439 | down | -1.5220 | CFLAR | CASP8 and FADD-like apoptosis regulator |
| 0.0223 | down | -1.5220 | MID1IP1 | MID1 interacting protein 1 )  transcript variant 1 |
| 0.0043 | down | -1.5224 | POLR1C | polymerase  I polypeptide C 30kDa  transcript variant 1 |
| 0.0066 | down | -1.5224 | CCT4 | chaperonin containing TCP1 subunit 4 |
| 0.0122 | down | -1.5225 | CPSF2 | cleavage and polyadenylation specific factor 2 100kDa |
| 0.0216 | down | -1.5235 | C20orf72 | chromosome 20 open reading frame 72 |
| 0.0052 | down | -1.5236 | GSTO2 | glutathione S-transferase omega 2 |
| 0.0302 | down | -1.5240 | CTSA | cathepsin A  transcript variant 1 |
| 0.0144 | down | -1.5241 | DHRS4 | dehydrogenase/reductase  member 4 |
| 0.0399 | down | -1.5250 | CHCHD10 | coiled-coil-helix-coiled-coil-helix domain containing 10 |
| 0.0194 | down | -1.5251 | SEH1L | SEH1-like   transcript variant 1 |
| 0.0476 | down | -1.5260 | AUH | AU RNA binding protein/enoyl-Coenzyme A hydratase  nuclear gene encoding mitochondrial protein |
| 0.0358 | down | -1.5260 | TK2 | thymidine kinase 2 mitochondrial  nuclear gene encoding mitochondrial protein |
| 0.0061 | down | -1.5267 | MRPL48 | mitochondrial ribosomal protein L48  nuclear gene encoding mitochondrial protein |
| 0.0269 | down | -1.5267 | SH3BGRL | SH3 domain binding glutamic acid-rich protein like |
| 0.0499 | down | -1.5273 | CALM3 | calmodulin 3 |
| 0.0032 | down | -1.5273 | ENOX2 | ecto-NOX disulfide-thiol exchanger 2  transcript variant 1 |
| 0.0146 | down | -1.5276 | LAGE3 | L antigen family member 3 |
| 0.0029 | down | -1.5281 | CSE1L | CSE1 chromosome segregation 1-like |
| 0.0077 | down | -1.5304 | PDSS1 | prenyl  diphosphate synthase subunit 1 |
| 0.0014 | down | -1.5309 | COPS7A | COP9 constitutive photomorphogenic homolog subunit 7A |
| 0.0088 | down | -1.5310 | ATP5I | ATP synthase H+ transporting mitochondrial F0 complex subunit E  nuclear gene encoding mitochondrial protein |
| 0.0214 | down | -1.5312 | SFXN2 | sideroflexin 2 |
| 0.0074 | down | -1.5317 | ALG8 | asparagine-linked glycosylation 8 homolog   transcript variant 1 |
| 0.0153 | down | -1.5324 | PCCA | propionyl Coenzyme A carboxylase alpha polypeptide  nuclear gene encoding mitochondrial protein transcript variant 1 |
| 0.0025 | down | -1.5324 | PCBD1 | pterin-4 alpha-carbinolamine dehydratase/dimerization cofactor of hepatocyte nuclear factor 1 alpha |
| 0.0175 | down | -1.5325 | GFM2 | G elongation factor mitochondrial 2  nuclear gene encoding mitochondrial protein transcript variant 3 |
| 0.0251 | down | -1.5325 | CCDC90B | coiled-coil domain containing 90B |
| 0.0077 | down | -1.5327 | C17orf61 | chromosome 17 open reading frame 61 |
| 0.0106 | down | -1.5335 | UFSP2 | UFM1-specific peptidase 2 |
| 0.0326 | down | -1.5342 | FAM173A | family with sequence similarity 173 member A |
| 0.0180 | down | -1.5346 | C11orf60 | chromosome 11 open reading frame 60 |
| 0.0146 | down | -1.5347 | OTUD6B | OTU domain containing 6B |
| 0.0310 | down | -1.5352 | CASP2 | caspase 2 apoptosis-related cysteine peptidase  transcript variant 1 |
| 0.0393 | down | -1.5352 | ADSS | adenylosuccinate synthase |
| 0.0007 | down | -1.5357 | MTIF2 | mitochondrial translational initiation factor 2  nuclear gene encoding mitochondrial protein transcript variant 1 |
| 0.0137 | down | -1.5359 | IMPA2 | inositol-1-monophosphatase 2 |
| 0.0299 | down | -1.5359 | TXNRD2 | thioredoxin reductase 2  nuclear gene encoding mitochondrial protein |
| 0.0273 | down | -1.5369 | SDHC | succinate dehydrogenase complex subunit C integral membrane protein 15kDa  nuclear gene encoding mitochondrial protein |
| 0.0265 | down | -1.5369 | UTP6 | UTP6 small subunit  processome component homolog |
| 0.0144 | down | -1.5375 | PFKFB2 | 6-phosphofructo-2-kinase/fructose-26-biphosphatase 2  transcript variant 1 |
| 0.0126 | down | -1.5375 | THADA | thyroid adenoma associated  transcript variant 1 |
| 0.0178 | down | -1.5379 | INTS9 | integrator complex subunit 9 |

| | | | | |
|---|---|---|---|---|
| 0.0189 | down | -1.5381 | SLC16A14 | solute carrier family 16 member 14 |
| 0.0319 | down | -1.5385 | HDDC3 | HD domain containing 3 |
| 0.0024 | down | -1.5386 | TATDN3 | TatD DNase domain containing 3 transcript variant 2 |
| 0.0103 | down | -1.5386 | CETN3 | centrin EF-hand protein 3 |
| 0.0265 | down | -1.5389 | PPIE | peptidylprolyl isomerase E transcript variant 3 |
| 0.0456 | down | -1.5390 | NOLA2 | nucleolar protein family A member 2 transcript variant 2 |
| 0.0217 | down | -1.5407 | LOC642197 | PREDICTED: similar to Protein FAM82B |
| 0.0214 | down | -1.5407 | ARSD | arylsulfatase D transcript variant 1 |
| 0.0441 | down | -1.5424 | SLC25A15 | solute carrier family 25 member 15 nuclear gene encoding mitochondrial protein |
| 0.0195 | down | -1.5425 | DHX36 | DEAH box polypeptide 36 |
| 0.0191 | down | -1.5430 | HSD17B4 | hydroxysteroid dehydrogenase 4 |
| 0.0454 | down | -1.5434 | ATP5J2 | ATP synthase H+ transporting mitochondrial F0 complex subunit F2 nuclear gene encoding mitochondrial protein transcript variant 1 |
| 0.0170 | down | -1.5434 | MS4A4A | membrane-spanning 4-domains subfamily A member 4 transcript variant 2 |
| 0.0181 | down | -1.5435 | NDUFA12 | NADH dehydrogenase 1 alpha subcomplex 12 |
| 0.0142 | down | -1.5439 | TMEM85 | transmembrane protein 85 |
| 0.0188 | down | -1.5440 | THYN1 | thymocyte nuclear protein 1 transcript variant 2 |
| 0.0322 | down | -1.5443 | TIMELESS | timeless homolog |
| 0.0150 | down | -1.5453 | FAM50B | family with sequence similarity 50 member B |
| 0.0035 | down | -1.5453 | BRP44L | brain protein 44-like |
| 0.0493 | down | -1.5457 | KLHL9 | kelch-like 9 |
| 0.0115 | down | -1.5458 | WDR67 | WD repeat domain 67 |
| 0.0085 | down | -1.5458 | DHRS4L2 | dehydrogenase/reductase member 4 like 2 |
| 0.0032 | down | -1.5461 | NDUFA7 | NADH dehydrogenase 1 alpha subcomplex 7 145kDa |
| 0.0423 | down | -1.5468 | TUFM | Tu translation elongation factor mitochondrial nuclear gene encoding mitochondrial protein |
| 0.0139 | down | -1.5473 | DHFRL1 | dihydrofolate reductase-like 1 |
| 0.0061 | down | -1.5474 | CMC1 | COX assembly mitochondrial protein homolog nuclear gene encoding mitochondrial protein |
| 0.0443 | down | -1.5475 | CRBN | cereblon |
| 0.0299 | down | -1.5482 | ANGEL1 | angel homolog 1 |
| 0.0002 | down | -1.5484 | NDUFV2 | NADH dehydrogenase flavoprotein 2 24kDa |
| 0.0487 | down | -1.5484 | NDUFAF2 | NADH dehydrogenase 1 alpha subcomplex assembly factor 2 |
| 0.0268 | down | -1.5484 | SUMF2 | sulfatase modifying factor 2 transcript variant 4 |
| 0.0133 | down | -1.5485 | MKKS | McKusick-Kaufman syndrome transcript variant 2 |
| 0.0164 | down | -1.5487 | TAF1B | TATA box binding protein -associated factor RNA polymerase I B 63kDa |
| 0.0482 | down | -1.5487 | MCAT | malonyl CoA:ACP acyltransferase nuclear gene encoding mitochondrial protein transcript variant 1 |
| 0.0452 | down | -1.5489 | C6orf211 | chromosome 6 open reading frame 211 |
| 0.0023 | down | -1.5490 | MTX2 | metaxin 2 transcript variant 1 |
| 0.0171 | down | -1.5498 | SKIV2L2 | superkiller viralicidic activity 2-like 2 |
| 0.0032 | down | -1.5498 | CHAC2 | ChaC cation transport regulator homolog 2 |
| 0.0005 | down | -1.5515 | UTP14A | UTP14 U3 small nucleolar ribonucleoprotein homolog A |
| 0.0481 | down | -1.5517 | PID1 | phosphotyrosine interaction domain containing 1 |
| 0.0346 | down | -1.5526 | EFEMP1 | EGF-containing fibulin-like extracellular matrix protein 1 transcript variant 1 |
| 0.0176 | down | -1.5534 | PHYH | phytanoyl-CoA 2-hydroxylase transcript variant 2 |
| 0.0168 | down | -1.5534 | TSPAN31 | tetraspanin 31 |
| 0.0330 | down | -1.5537 | DYNC2LI1 | dynein cytoplasmic 2 light intermediate chain 1 transcript variant 1 |
| 0.0188 | down | -1.5537 | THAP10 | THAP domain containing 10 |
| 0.0446 | down | -1.5540 | HINT2 | histidine triad nucleotide binding protein 2 |
| 0.0069 | down | -1.5540 | AURKA | aurora kinase A transcript variant 5 |

| | | | | |
|---|---|---|---|---|
| 0.0094 | down | -1.5541 | WRB | tryptophan rich basic protein |
| 0.0109 | down | -1.5543 | ARMC10 | armadillo repeat containing 10 |
| 0.0365 | down | -1.5544 | C5orf28 | chromosome 5 open reading frame 28 |
| 0.0478 | down | -1.5545 | ZMAT5 | zinc finger matrin type 5 transcript variant 1 |
| 0.0136 | down | -1.5545 | LOC134145 | hypothetical protein LOC134145 |
| 0.0373 | down | -1.5552 | C4orf41 | chromosome 4 open reading frame 41 transcript variant 1 |
| 0.0323 | down | -1.5555 | TIAL1 | TIA1 cytotoxic granule-associated RNA binding protein-like 1 transcript variant 1 |
| 0.0280 | down | -1.5561 | | cDNA FLJ13267 fis clone OVARC1000964 |
| 0.0058 | down | -1.5567 | JTV1 | JTV1 gene |
| 0.0059 | down | -1.5572 | ATPBD4 | ATP binding domain 4 |
| 0.0021 | down | -1.5573 | C1orf97 | chromosome 1 open reading frame 97 |
| 0.0149 | down | -1.5588 | LOC644310 | PREDICTED: similar to ubiquinol-cytochrome c reductase complex |
| 0.0269 | down | -1.5596 | BBS4 | Bardet-Biedl syndrome 4 |
| 0.0134 | down | -1.5596 | RNF14 | ring finger protein 14 transcript variant 4 |
| 0.0138 | down | -1.5597 | BRP44L | brain protein 44-like |
| 0.0082 | down | -1.5601 | SNX2 | sorting nexin 2 |
| 0.0203 | down | -1.5603 | C14orf104 | chromosome 14 open reading frame 104 transcript variant 1 |
| 0.0159 | down | -1.5606 | MKI67IP | MKI67 interacting nucleolar phosphoprotein |
| 0.0331 | down | -1.5606 | EIF2S2 | eukaryotic translation initiation factor 2 subunit 2 beta 38kDa |
| 0.0166 | down | -1.5618 | B3GALNT1 | beta-13-N-acetylgalactosaminyltransferase 1 transcript variant 3 |
| 0.0185 | down | -1.5623 | MRPS28 | mitochondrial ribosomal protein S28 nuclear gene encoding mitochondrial protein |
| 0.0206 | down | -1.5623 | LOC654085 | PREDICTED: similar to Glycine cleavage system H protein mitochondrial precursor transcript variant 1 |
| 0.0104 | down | -1.5627 | C6orf66 | chromosome 6 open reading frame 66 |
| 0.0301 | down | -1.5631 | MRPL19 | mitochondrial ribosomal protein L19 nuclear gene encoding mitochondrial protein |
| 0.0292 | down | -1.5637 | TXNDC10 | thioredoxin domain containing 10 |
| 0.0450 | down | -1.5642 | LPL | lipoprotein lipase |
| 0.0261 | down | -1.5645 | C1orf97 | chromosome 1 open reading frame 97 |
| 0.0153 | down | -1.5648 | NDFIP1 | Nedd4 family interacting protein 1 |
| 0.0100 | down | -1.5651 | SPATA20 | spermatogenesis associated 20 |
| 0.0180 | down | -1.5652 | SUGT1 | SGT1 suppressor of G2 allele of SKP1 |
| 0.0139 | down | -1.5656 | ASH2L | ash2 -like |
| 0.0322 | down | -1.5657 | C1orf57 | chromosome 1 open reading frame 57 |
| 0.0412 | down | -1.5660 | C1QBP | complement component 1 q subcomponent binding protein nuclear gene encoding mitochondrial protein |
| 0.0036 | down | -1.5661 | GTF2H5 | general transcription factor IIH polypeptide 5 |
| 0.0142 | down | -1.5665 | AASDHPPT | aminoadipate-semialdehyde dehydrogenase-phosphopantetheinyl transferase |
| 0.0094 | down | -1.5671 | LYAR | Ly1 antibody reactive homolog |
| 0.0283 | down | -1.5674 | ACN9 | ACN9 homolog |
| 0.0391 | down | -1.5675 | LOC730024 | PREDICTED: similar to male sterility domain containing 1 |
| 0.0461 | down | -1.5678 | C9orf95 | chromosome 9 open reading frame 95 |
| 0.0039 | down | -1.5682 | MRPL18 | mitochondrial ribosomal protein L18 nuclear gene encoding mitochondrial protein |
| 0.0183 | down | -1.5684 | APRT | adenine phosphoribosyltransferase transcript variant 1 |
| 0.0333 | down | -1.5704 | FBXO6 | F-box protein 6 |
| 0.0272 | down | -1.5710 | LRRC1 | leucine rich repeat containing 1 |
| 0.0258 | down | -1.5711 | C2orf43 | chromosome 2 open reading frame 43 |
| 0.0123 | down | -1.5728 | WDR7 | WD repeat domain 7 transcript variant 1 |
| 0.0174 | down | -1.5743 | ALS2CR4 | amyotrophic lateral sclerosis 2 chromosome region candidate 4 transcript variant 1 |
| 0.0101 | down | -1.5744 | CCDC90B | coiled-coil domain containing 90B |

| | | | | |
|---|---|---|---|---|
| 0.0365 | down | -1.5752 | TCHP | trichoplein keratin filament binding |
| 0.0398 | down | -1.5753 | C4orf41 | chromosome 4 open reading frame 41  transcript variant 2 |
| 0.0241 | down | -1.5757 | PRELID1 | PRELI domain containing 1 |
| 0.0012 | down | -1.5761 | ZNF828 | zinc finger protein 828 |
| 0.0008 | down | -1.5762 | SCO1 | SCO cytochrome oxidase deficient homolog 1   nuclear gene encoding mitochondrial protein |
| 0.0426 | down | -1.5768 | PSME2 | proteasome  activator subunit 2 |
| 0.0163 | down | -1.5780 | LPCAT3 | lysophosphatidylcholine acyltransferase 3 |
| 0.0226 | down | -1.5780 | ATP5D | ATP synthase H+ transporting mitochondrial F1 complex delta subunit  nuclear gene encoding mitochondrial protein transcript variant 1 |
| 0.0315 | down | -1.5801 | TNFAIP6 | tumor necrosis factor alpha-induced protein 6 |
| 0.0102 | down | -1.5807 | PTPLB | protein tyrosine phosphatase-like  member b |
| 0.0039 | down | -1.5814 | C7orf44 | chromosome 7 open reading frame 44 |
| 0.0112 | down | -1.5832 | C11orf59 | chromosome 11 open reading frame 59 |
| 0.0180 | down | -1.5839 | PARP9 | poly  polymerase family member 9 |
| 0.0057 | down | -1.5859 | TRIM68 | tripartite motif-containing 68 |
| 0.0133 | down | -1.5863 | MRPL27 | mitochondrial ribosomal protein L27  nuclear gene encoding mitochondrial protein transcript variant 2 |
| 0.0057 | down | -1.5864 | LGMN | legumain  transcript variant 2 |
| 0.0021 | down | -1.5874 | PSMB10 | proteasome  subunit beta type 10 |
| 0.0079 | down | -1.5876 | COQ6 | coenzyme Q6 homolog monooxygenase   transcript variant 1 |
| 0.0484 | down | -1.5883 | CCDC44 | coiled-coil domain containing 44 |
| 0.0077 | down | -1.5886 | MSTO1 | misato homolog 1 |
| 0.0144 | down | -1.5903 | CCDC53 | coiled-coil domain containing 53 |
| 0.0438 | down | -1.5903 | OSGEPL1 | O-sialoglycoprotein endopeptidase-like 1 |
| 0.0228 | down | -1.5917 | MRPS27 | mitochondrial ribosomal protein S27  nuclear gene encoding mitochondrial protein |
| 0.0030 | down | -1.5918 | MRPL34 | mitochondrial ribosomal protein L34  nuclear gene encoding mitochondrial protein |
| 0.0410 | down | -1.5921 | GPS1 | G protein pathway suppressor 1  transcript variant 2 |
| 0.0336 | down | -1.5921 | MITD1 | MIT microtubule interacting and transport domain containing 1 |
| 0.0425 | down | -1.5928 | PCYT2 | phosphate cytidylyltransferase 2 ethanolamine |
| 0.0116 | down | -1.5931 | FAM86A | family with sequence similarity 86 member A  transcript variant 2 |
| 0.0398 | down | -1.5936 | MFAP1 | microfibrillar-associated protein 1 |
| 0.0288 | down | -1.5941 | TMEM60 | transmembrane protein 60 |
| 0.0307 | down | -1.5957 | UQCC | ubiquinol-cytochrome c reductase complex chaperone  transcript variant 1 |
| 0.0339 | down | -1.5967 | TMEM175 | transmembrane protein 175 |
| 0.0058 | down | -1.5968 | AURKA | aurora kinase A  transcript variant 3 |
| 0.0154 | down | -1.5974 | ZBTB9 | zinc finger and BTB domain containing 9 |
| 0.0427 | down | -1.5975 | LARS2 | leucyl-tRNA synthetase 2 mitochondrial  nuclear gene encoding mitochondrial protein |
| 0.0288 | down | -1.5977 | APOA1BP | apolipoprotein A-I binding protein |
| 0.0326 | down | -1.5981 | ISOC2 | isochorismatase domain containing 2 |
| 0.0051 | down | -1.5982 | DPH2 | DPH2 homolog   transcript variant 1 |
| 0.0243 | down | -1.5987 | PRIM1 | primase DNA polypeptide 1 |
| 0.0232 | down | -1.5989 | EPHX2 | epoxide hydrolase 2 cytoplasmic |
| 0.0373 | down | -1.5993 | AGTRAP | angiotensin II receptor-associated protein  transcript variant 2 |
| 0.0170 | down | -1.6003 | CKAP5 | cytoskeleton associated protein 5  transcript variant 1 |
| 0.0493 | down | -1.6015 | TRAPPC6B | trafficking protein particle complex 6B  transcript variant 1 |
| 0.0002 | down | -1.6018 | POLR3K | polymerase  III  polypeptide K 123 kDa |
| 0.0334 | down | -1.6020 | PTPRE | protein tyrosine phosphatase receptor type E  transcript variant 2 |
| 0.0147 | down | -1.6023 | FAHD2A | fumarylacetoacetate hydrolase domain containing 2A |
| 0.0271 | down | -1.6024 | CPSF2 | cleavage and polyadenylation specific factor 2 100kDa |

| | | | | |
|---|---|---|---|---|
| 0.0388 | down | -1.6029 | ZWILCH | Zwilch kinetochore associated homolog  transcript variant 2 transcribed RNA |
| 0.0204 | down | -1.6030 | ATP5F1 | ATP synthase H+ transporting mitochondrial F0 complex subunit B1  nuclear gene encoding mitochondrial protein |
| 0.0357 | down | -1.6037 | ZNF187 | zinc finger protein 187  transcript variant 1 |
| 0.0116 | down | -1.6039 | AHCY | S-adenosylhomocysteine hydrolase |
| 0.0083 | down | -1.6041 | POLR1C | polymerase  I polypeptide C 30kDa  transcript variant 1 |
| 0.0327 | down | -1.6041 | GFM1 | G elongation factor mitochondrial 1  nuclear gene encoding mitochondrial protein |
| 0.0278 | down | -1.6048 | PIGU | phosphatidylinositol glycan anchor biosynthesis class U |
| 0.0176 | down | -1.6053 | CUL5 | cullin 5 |
| 0.0062 | down | -1.6054 | LYAR | Ly1 antibody reactive homolog |
| 0.0368 | down | -1.6056 | PSMG1 | proteasome  assembly chaperone 1  transcript variant 1 |
| 0.0381 | down | -1.6056 | BCAS4 | breast carcinoma amplified sequence 4  transcript variant 1 |
| 0.0355 | down | -1.6056 | PSMB9 | proteasome  subunit beta type 9   transcript variant 1 |
| 0.0068 | down | -1.6060 | C20orf30 | chromosome 20 open reading frame 30  transcript variant 1 |
| 0.0170 | down | -1.6069 | RAB31 | RAB31 member RAS oncogene family |
| 0.0348 | down | -1.6076 | BZW2 | basic leucine zipper and W2 domains 2 |
| 0.0245 | down | -1.6083 | HRSP12 | heat-responsive protein 12 |
| 0.0012 | down | -1.6084 | PCCB | propionyl Coenzyme A carboxylase beta polypeptide |
| 0.0283 | down | -1.6091 | FDFT1 | farnesyl-diphosphate farnesyltransferase 1 |
| 0.0011 | down | -1.6097 | HDDC2 | HD domain containing 2 |
| 0.0072 | down | -1.6098 | DLD | dihydrolipoamide dehydrogenase |
| 0.0236 | down | -1.6103 | C1orf123 | chromosome 1 open reading frame 123 |
| 0.0006 | down | -1.6105 | MRPL18 | mitochondrial ribosomal protein L18  nuclear gene encoding mitochondrial protein |
| 0.0141 | down | -1.6110 | LOC54103 | hypothetical protein LOC54103 |
| 0.0216 | down | -1.6111 | PTPLAD1 | protein tyrosine phosphatase-like A domain containing 1 |
| 0.0177 | down | -1.6112 | NSUN5 | NOL1/NOP2/Sun domain family member 5  transcript variant 1 |
| 0.0054 | down | -1.6112 | | ; cDNA DKFZp779M2422 |
| 0.0211 | down | -1.6115 | C20orf27 | chromosome 20 open reading frame 27 |
| 0.0008 | down | -1.6116 | CCT6A | chaperonin containing TCP1 subunit 6A   transcript variant 2 |
| 0.0025 | down | -1.6116 | C1orf181 | chromosome 1 open reading frame 181 |
| 0.0440 | down | -1.6116 | ERLIN2 | ER lipid raft associated 2  transcript variant 1 |
| 0.0201 | down | -1.6120 | CLUAP1 | clusterin associated protein 1  transcript variant 1 |
| 0.0344 | down | -1.6123 | STAMBP | STAM binding protein  transcript variant 1 |
| 0.0007 | down | -1.6124 | FAM96A | family with sequence similarity 96 member A  transcript variant 2 |
| 0.0058 | down | -1.6124 | MRPL38 | mitochondrial ribosomal protein L38  nuclear gene encoding mitochondrial protein |
| 0.0411 | down | -1.6128 | MRPL12 | mitochondrial ribosomal protein L12  nuclear gene encoding mitochondrial protein |
| 0.0266 | down | -1.6144 | CYBASC3 | cytochrome b ascorbate dependent 3 |
| 0.0119 | down | -1.6144 | ZNF239 | zinc finger protein 239  transcript variant 4 |
| 0.0162 | down | -1.6144 | APRT | adenine phosphoribosyltransferase  transcript variant 1 |
| 0.0132 | down | -1.6151 | NMI | N-myc  interactor |
| 0.0215 | down | -1.6151 | RSBN1L | round spermatid basic protein 1-like |
| 0.0241 | down | -1.6154 | CLYBL | citrate lyase beta like |
| 0.0136 | down | -1.6156 | C9orf23 | chromosome 9 open reading frame 23  transcript variant 1 |
| 0.0282 | down | -1.6158 | ARPC1B | actin related protein 2/3 complex subunit 1B 41kDa |
| 0.0305 | down | -1.6166 | THYN1 | thymocyte nuclear protein 1  transcript variant 3 |
| 0.0236 | down | -1.6169 | TMTC4 | transmembrane and tetratricopeptide repeat containing 4  transcript variant 2 |
| 0.0485 | down | -1.6170 | ARFIP1 | ADP-ribosylation factor interacting protein 1  transcript variant 2 |
| 0.0190 | down | -1.6173 | C17orf90 | chromosome 17 open reading frame 90 |

| | | | | |
|---|---|---|---|---|
| 0.0230 | down | -1.6185 | MID1IP1 | MID1 interacting protein 1 ) |
| 0.0300 | down | -1.6186 | ACACA | acetyl-Coenzyme A carboxylase alpha  transcript variant 2 |
| 0.0160 | down | -1.6192 | ADCK1 | aarF domain containing kinase 1 |
| 0.0343 | down | -1.6194 | ARHGAP18 | Rho GTPase activating protein 18 |
| 0.0238 | down | -1.6195 | RABL4 | RAB member of RAS oncogene family-like 4 |
| 0.0219 | down | -1.6196 | PGD | phosphogluconate dehydrogenase |
| 0.0004 | down | -1.6201 | MRPL13 | mitochondrial ribosomal protein L13  nuclear gene encoding mitochondrial protein |
| 0.0411 | down | -1.6206 | CTSC | cathepsin C  transcript variant 1 |
| 0.0173 | down | -1.6212 | PUS1 | pseudouridylate synthase 1  transcript variant 2 |
| 0.0070 | down | -1.6212 | CPT2 | carnitine palmitoyltransferase II  nuclear gene encoding mitochondrial protein |
| 0.0327 | down | -1.6217 | HMBS | hydroxymethylbilane synthase  transcript variant 1 |
| 0.0211 | down | -1.6219 | MANEA | mannosidase endo-alpha |
| 0.0412 | down | -1.6222 | C11orf67 | chromosome 11 open reading frame 67 |
| 0.0285 | down | -1.6223 | MFF | mitochondrial fission factor  nuclear gene encoding mitochondrial protein |
| 0.0038 | down | -1.6225 | PDHA1 | pyruvate dehydrogenase  alpha 1 |
| 0.0063 | down | -1.6231 | CPSF3 | cleavage and polyadenylation specific factor 3 73kDa |
| 0.0164 | down | -1.6240 | TMEM69 | transmembrane protein 69 |
| 0.0089 | down | -1.6247 | CCDC99 | coiled-coil domain containing 99 |
| 0.0456 | down | -1.6251 | UBE2L3 | ubiquitin-conjugating enzyme E2L 3  transcript variant 2 |
| 0.0152 | down | -1.6257 | C11orf74 | chromosome 11 open reading frame 74 |
| 0.0130 | down | -1.6258 | LSM4 | LSM4 homolog U6 small nuclear RNA associated |
| 0.0484 | down | -1.6261 | N6AMT2 | N-6 adenine-specific DNA methyltransferase 2 |
| 0.0351 | down | -1.6270 | ELOVL6 | ELOVL family member 6 elongation of long chain fatty acids |
| 0.0382 | down | -1.6271 | PQLC3 | PQ loop repeat containing 3 |
| 0.0134 | down | -1.6272 | GPAM | glycerol-3-phosphate acyltransferase mitochondrial |
| 0.0044 | down | -1.6272 | SNAPC5 | small nuclear RNA activating complex polypeptide 5 19kDa |
| 0.0198 | down | -1.6276 | CHCHD1 | coiled-coil-helix-coiled-coil-helix domain containing 1 |
| 0.0316 | down | -1.6278 | DPH5 | DPH5 homolog   transcript variant 1 |
| 0.0376 | down | -1.6283 | C11orf75 | chromosome 11 open reading frame 75 |
| 0.0114 | down | -1.6291 | MINA | MYC induced nuclear antigen  transcript variant 2 |
| 0.0201 | down | -1.6292 | WRB | tryptophan rich basic protein |
| 0.0194 | down | -1.6296 | C8orf38 | chromosome 8 open reading frame 38 |
| 0.0024 | down | -1.6297 | MSRB2 | methionine sulfoxide reductase B2 |
| 0.0161 | down | -1.6325 | HRSP12 | heat-responsive protein 12 |
| 0.0416 | down | -1.6335 | COQ3 | coenzyme Q3 homolog methyltransferase |
| 0.0006 | down | -1.6341 | PPA2 | pyrophosphatase  2  nuclear gene encoding mitochondrial protein transcript variant 3 |
| 0.0106 | down | -1.6352 | LIPT1 | lipoyltransferase 1  transcript variant 1 |
| 0.0054 | down | -1.6360 | RNGTT | RNA guanylyltransferase and 5'-phosphatase |
| 0.0185 | down | -1.6365 | LYCAT | lysocardiolipin acyltransferase  transcript variant 2 |
| 0.0126 | down | -1.6368 | PPAP2B | phosphatidic acid phosphatase type 2B  transcript variant 1 |
| 0.0360 | down | -1.6371 | C5orf44 | chromosome 5 open reading frame 44  transcript variant 3 |
| 0.0059 | down | -1.6371 | ICT1 | immature colon carcinoma transcript 1 |
| 0.0353 | down | -1.6373 | ACSL1 | acyl-CoA synthetase long-chain family member 1 |
| 0.0029 | down | -1.6374 | NDUFAF1 | NADH dehydrogenase  1 alpha subcomplex assembly factor 1 |
| 0.0006 | down | -1.6375 | C11orf48 | chromosome 11 open reading frame 48 |
| 0.0343 | down | -1.6377 | C17orf70 | chromosome 17 open reading frame 70 |
| 0.0146 | down | -1.6378 | ZMPSTE24 | zinc metallopeptidase |

| | | | | |
|---|---|---|---|---|
| 0.0042 | down | -1.6383 | DGCR6 | DiGeorge syndrome critical region gene 6 |
| 0.0114 | down | -1.6387 | EEF1B2 | eukaryotic translation elongation factor 1 beta 2 transcript variant 1 |
| 0.0278 | down | -1.6400 | MORN4 | MORN repeat containing 4 transcript variant 1 |
| 0.0123 | down | -1.6401 | G6PD | glucose-6-phosphate dehydrogenase transcript variant 1 |
| 0.0438 | down | -1.6404 | NCBP1 | nuclear cap binding protein subunit 1 80kDa |
| 0.0325 | down | -1.6406 | FAM173B | family with sequence similarity 173 member B |
| 0.0018 | down | -1.6409 | LSM5 | LSM5 homolog U6 small nuclear RNA associated |
| 0.0014 | down | -1.6410 | APEX1 | APEX nuclease 1 transcript variant 1 |
| 0.0013 | down | -1.6410 | DGCR6 | DiGeorge syndrome critical region gene 6 |
| 0.0295 | down | -1.6411 | LOC653513 | PREDICTED: similar to phosphodiesterase 4D interacting protein isoform 2 |
| 0.0182 | down | -1.6412 | AP3M1 | adaptor-related protein complex 3 mu 1 subunit transcript variant 1 |
| 0.0176 | down | -1.6415 | PSMB8 | proteasome subunit beta type 8 transcript variant 1 |
| 0.0287 | down | -1.6423 | RABEP1 | rabaptin RAB GTPase binding effector protein 1 transcript variant 2 |
| 0.0087 | down | -1.6435 | FLJ38482 | hypothetical protein FLJ38482 |
| 0.0006 | down | -1.6444 | LSM5 | LSM5 homolog U6 small nuclear RNA associated |
| 0.0008 | down | -1.6446 | EXOSC1 | exosome component 1 XM_944315 XM_944318 XM_944323 |
| 0.0046 | down | -1.6447 | RPAP3 | RNA polymerase II associated protein 3 |
| 0.0394 | down | -1.6450 | NTHL1 | nth endonuclease III-like 1 |
| 0.0065 | down | -1.6461 | TTC5 | tetratricopeptide repeat domain 5 |
| 0.0412 | down | -1.6467 | KIAA0196 | KIAA0196 |
| 0.0002 | down | -1.6471 | FAM96A | family with sequence similarity 96 member A transcript variant 1 |
| 0.0352 | down | -1.6473 | KRIT1 | KRIT1 ankyrin repeat containing transcript variant 3 |
| 0.0317 | down | -1.6478 | MTFR1 | mitochondrial fission regulator 1 nuclear gene encoding mitochondrial protein |
| 0.0416 | down | -1.6478 | TYSND1 | trypsin domain containing 1 transcript variant 2 |
| 0.0174 | down | -1.6490 | NDUFB6 | NADH dehydrogenase 1 beta subcomplex 6 17kDa nuclear gene encoding mitochondrial protein transcript variant 1 |
| 0.0085 | down | -1.6503 | MRPL11 | mitochondrial ribosomal protein L11 nuclear gene encoding mitochondrial protein transcript variant 1 |
| 0.0486 | down | -1.6504 | NIN | ninein transcript variant 2 |
| 0.0245 | down | -1.6505 | MTRR | 5-methyltetrahydrofolate-homocysteine methyltransferase reductase transcript variant 2 |
| 0.0298 | down | -1.6510 | IFIH1 | interferon induced with helicase C domain 1 |
| 0.0229 | down | -1.6515 | BRD8 | bromodomain containing 8 transcript variant 3 |
| 0.0094 | down | -1.6519 | DDX52 | DEAD box polypeptide 52 transcript variant 1 |
| 0.0122 | down | -1.6523 | C7orf11 | chromosome 7 open reading frame 11 |
| 0.0370 | down | -1.6526 | APOE | apolipoprotein E |
| 0.0008 | down | -1.6537 | NDUFS8 | NADH dehydrogenase Fe-S protein 8 23kDa |
| 0.0102 | down | -1.6550 | SSBP1 | single-stranded DNA binding protein 1 |
| 0.0291 | down | -1.6552 | FARS2 | phenylalanyl-tRNA synthetase 2 mitochondrial nuclear gene encoding mitochondrial protein |
| 0.0049 | down | -1.6579 | CLDN23 | claudin 23 |
| 0.0111 | down | -1.6587 | NAPRT1 | nicotinate phosphoribosyltransferase domain containing 1 |
| 0.0052 | down | -1.6590 | MRPL21 | mitochondrial ribosomal protein L21 nuclear gene encoding mitochondrial protein transcript variant 1 |
| 0.0055 | down | -1.6608 | C2orf76 | chromosome 2 open reading frame 76 |
| 0.0492 | down | -1.6609 | | cDNA clone IMAGE:5263177 |
| 0.0185 | down | -1.6613 | AGTRAP | angiotensin II receptor-associated protein transcript variant 4 |
| 0.0067 | down | -1.6618 | APEX1 | APEX nuclease 1 transcript variant 3 |
| 0.0228 | down | -1.6627 | LOC400506 | similar to TSG1181 |
| 0.0023 | down | -1.6629 | ACTR6 | ARP6 actin-related protein 6 homolog |
| 0.0062 | down | -1.6634 | ZNF684 | zinc finger protein 684 |
| 0.0482 | down | -1.6639 | AP1B1 | adaptor-related protein complex 1 beta 1 subunit transcript variant 2 |

| | | | | |
|---|---|---|---|---|
| 0.0042 | down | -1.6640 | C9orf142 | chromosome 9 open reading frame 142 |
| 0.0274 | down | -1.6642 | SLC5A6 | solute carrier family 5  member 6 |
| 0.0199 | down | -1.6644 | ZNF30 | zinc finger protein 30  transcript variant 2 |
| 0.0435 | down | -1.6648 | C9orf45 | PREDICTED:  chromosome 9 open reading frame 45  misc RNA |
| 0.0356 | down | -1.6652 | KIAA0859 | KIAA0859  transcript variant 3 |
| 0.0065 | down | -1.6656 | C18orf55 | chromosome 18 open reading frame 55 |
| 0.0250 | down | -1.6659 | HNMT | histamine N-methyltransferase  transcript variant 2 |
| 0.0469 | down | -1.6669 | SACM1L | SAC1 suppressor of actin mutations 1-like |
| 0.0031 | down | -1.6671 | RFC4 | replication factor C  4 37kDa  transcript variant 1 |
| 0.0090 | down | -1.6675 | MRPL46 | mitochondrial ribosomal protein L46  nuclear gene encoding mitochondrial protein |
| 0.0089 | down | -1.6679 | TTL | tubulin tyrosine ligase |
| 0.0306 | down | -1.6680 | STRADA | STE20-related kinase adaptor alpha  transcript variant 2 |
| 0.0120 | down | -1.6683 | CCDC76 | coiled-coil domain containing 76 |
| 0.0072 | down | -1.6686 | ZNF331 | zinc finger protein 331  transcript variant 1 |
| 0.0308 | down | -1.6690 | CSTF2T | cleavage stimulation factor 3' pre-RNA subunit 2 64kDa tau variant |
| 0.0066 | down | -1.6692 | FAM125A | family with sequence similarity 125 member A |
| 0.0109 | down | -1.6695 | STRBP | spermatid perinuclear RNA binding protein |
| 0.0314 | down | -1.6704 | SDHC | succinate dehydrogenase complex subunit C integral membrane protein 15kDa  nuclear gene encoding mitochondrial protein transcript variant 4 |
| 0.0194 | down | -1.6719 | MMACHC | methylmalonic aciduria  cblC type with homocystinuria |
| 0.0082 | down | -1.6722 | IDI1 | isopentenyl-diphosphate delta isomerase 1 |
| 0.0420 | down | -1.6723 | MRPS26 | mitochondrial ribosomal protein S26  nuclear gene encoding mitochondrial protein |
| 0.0096 | down | -1.6728 | EMG1 | EMG1 nucleolar protein homolog |
| 0.0353 | down | -1.6730 | TIPRL | TIP41 TOR signaling pathway regulator-like   transcript variant 1 |
| 0.0248 | down | -1.6739 | AP3M1 | adaptor-related protein complex 3 mu 1 subunit  transcript variant 2 |
| 0.0466 | down | -1.6740 | PIGN | phosphatidylinositol glycan anchor biosynthesis class N  transcript variant 1 |
| 0.0466 | down | -1.6747 | RENBP | renin binding protein |
| 0.0246 | down | -1.6748 | MIPEP | mitochondrial intermediate peptidase  nuclear gene encoding mitochondrial protein |
| 0.0095 | down | -1.6750 | PCBD1 | pterin-4 alpha-carbinolamine dehydratase/dimerization cofactor of hepatocyte nuclear factor 1 alpha |
| 0.0105 | down | -1.6755 | TXNDC14 | thioredoxin domain containing 14 |
| 0.0068 | down | -1.6759 | RAD51C | RAD51 homolog C   transcript variant 2 |
| 0.0112 | down | -1.6759 | GOT2 | glutamic-oxaloacetic transaminase 2 mitochondrial   nuclear gene encoding mitochondrial protein |
| 0.0086 | down | -1.6765 | SPSB2 | splA/ryanodine receptor domain and SOCS box containing 2 |
| 0.0225 | down | -1.6765 | ATPAF1 | ATP synthase mitochondrial F1 complex assembly factor 1  nuclear gene encoding mitochondrial protein transcript variant 1 |
| 0.0234 | down | -1.6776 | RCC2 | regulator of chromosome condensation 2 |
| 0.0325 | down | -1.6784 | SAC3D1 | SAC3 domain containing 1 |
| 0.0075 | down | -1.6785 | C12orf62 | chromosome 12 open reading frame 62 |
| 0.0138 | down | -1.6792 | NAP1L4 | nucleosome assembly protein 1-like 4 |
| 0.0478 | down | -1.6793 | ZDHHC16 | zinc finger DHHC-type containing 16  transcript variant 1 |
| 0.0015 | down | -1.6795 | RNASEH2B | ribonuclease H2 subunit B |
| 0.0189 | down | -1.6795 | C6orf203 | chromosome 6 open reading frame 203 |
| 0.0418 | down | -1.6797 | EXOSC3 | exosome component 3  transcript variant 1 |
| 0.0116 | down | -1.6812 | LOC653308 | PREDICTED:  similar to N-acylsphingosine amidohydrolase 2 transcript variant 1 |
| 0.0221 | down | -1.6813 | TMEM116 | transmembrane protein 116 |
| 0.0390 | down | -1.6817 | DHFRL1 | dihydrofolate reductase-like 1 |
| 0.0152 | down | -1.6828 | MTMR9 | myotubularin related protein 9 |
| 0.0039 | down | -1.6828 | ALDH9A1 | aldehyde dehydrogenase 9 family member A1 |
| 0.0032 | down | -1.6829 | MTHFS | 510-methenyltetrahydrofolate synthetase |

| | | | | |
|---|---|---|---|---|
| 0.0020 | down | -1.6829 | RSAD1 | radical S-adenosyl methionine domain containing 1 |
| 0.0343 | down | -1.6842 | PRIM1 | primase DNA polypeptide 1 |
| 0.0142 | down | -1.6878 | SPR | sepiapterin reductase |
| 0.0281 | down | -1.6878 | MRPS11 | mitochondrial ribosomal protein S11  nuclear gene encoding mitochondrial protein transcript variant 2 |
| 0.0265 | down | -1.6880 | APTX | aprataxin  transcript variant 5 |
| 0.0015 | down | -1.6890 | SUCLG1 | succinate-CoA ligase alpha subunit |
| 0.0421 | down | -1.6890 | CD14 | CD14 molecule  transcript variant 1 |
| 0.0155 | down | -1.6902 | DCN | decorin  transcript variant A2 |
| 0.0496 | down | -1.6916 | FASTKD1 | FAST kinase domains 1 |
| 0.0105 | down | -1.6919 | MPHOSPH6 | M-phase phosphoprotein 6 |
| 0.0117 | down | -1.6937 | KIAA0564 | KIAA0564  transcript variant 1 |
| 0.0281 | down | -1.6963 | CAT | catalase |
| 0.0014 | down | -1.6964 | C6orf125 | chromosome 6 open reading frame 125 |
| 0.0485 | down | -1.6968 | UCP2 | uncoupling protein 2   nuclear gene encoding mitochondrial protein |
| 0.0093 | down | -1.6976 | C14orf179 | chromosome 14 open reading frame 179 |
| 0.0083 | down | -1.6979 | MCEE | methylmalonyl CoA epimerase |
| 0.0076 | down | -1.6983 | HIBADH | 3-hydroxyisobutyrate dehydrogenase |
| 0.0097 | down | -1.6985 | TSPAN13 | tetraspanin 13 |
| 0.0214 | down | -1.6998 | C6orf130 | chromosome 6 open reading frame 130 |
| 0.0396 | down | -1.6998 | NAGK | N-acetylglucosamine kinase |
| 0.0279 | down | -1.7007 | SLC25A20 | solute carrier family 25  member 20  nuclear gene encoding mitochondrial protein |
| 0.0016 | down | -1.7008 | AIFM1 | apoptosis-inducing factor mitochondrion-associated 1  nuclear gene encoding mitochondrial protein transcript variant 3 |
| 0.0118 | down | -1.7011 | TM2D1 | TM2 domain containing 1 |
| 0.0251 | down | -1.7011 | WDR18 | WD repeat domain 18 |
| 0.0161 | down | -1.7018 | BANF1 | barrier to autointegration factor 1 |
| 0.0013 | down | -1.7018 | PIGK | phosphatidylinositol glycan anchor biosynthesis class K |
| 0.0019 | down | -1.7025 | CYC1 | cytochrome c-1 |
| 0.0269 | down | -1.7031 | DLAT | dihydrolipoamide S-acetyltransferase |
| 0.0056 | down | -1.7032 | AGK | acylglycerol kinase |
| 0.0119 | down | -1.7047 | PAQR3 | progestin and adipoQ receptor family member III |
| 0.0425 | down | -1.7053 | DNCL1 | dynein cytoplasmic light polypeptide 1 |
| 0.0041 | down | -1.7055 | HADHB | hydroxyacyl-Coenzyme A dehydrogenase/3-ketoacyl-Coenzyme A thiolase/enoyl-Coenzyme A hydratase  beta subunit  nuclear gene encoding mitochondrial protein |
| 0.0117 | down | -1.7075 | LANCL1 | LanC lantibiotic synthetase component C-like 1 |
| 0.0357 | down | -1.7081 | ZBED5 | zinc finger BED-type containing 5 |
| 0.0262 | down | -1.7084 | PAFAH2 | platelet-activating factor acetylhydrolase 2 40kDa |
| 0.0353 | down | -1.7090 | TGM2 | transglutaminase 2   transcript variant 1 |
| 0.0146 | down | -1.7090 | VRK3 | vaccinia related kinase 3  transcript variant 2 |
| 0.0404 | down | -1.7092 | C1orf56 | chromosome 1 open reading frame 56 |
| 0.0093 | down | -1.7094 | LIPT1 | lipoyltransferase 1  transcript variant 1 |
| 0.0140 | down | -1.7120 | ALDH7A1 | aldehyde dehydrogenase 7 family member A1 |
| 0.0001 | down | -1.7122 | ETFA | electron-transfer-flavoprotein alpha polypeptide   nuclear gene encoding mitochondrial protein |
| 0.0495 | down | -1.7123 | NME3 | non-metastatic cells 3 protein expressed in |
| 0.0123 | down | -1.7134 | KLHL12 | kelch-like 12 |
| 0.0062 | down | -1.7134 | ASPSCR1 | alveolar soft part sarcoma chromosome region candidate 1 |
| 0.0122 | down | -1.7144 | FKBP3 | FK506 binding protein 3 25kDa |
| 0.0355 | down | -1.7145 | C2orf64 | chromosome 2 open reading frame 64 |
| 0.0345 | down | -1.7146 | PDHB | pyruvate dehydrogenase  beta |

| | | | | |
|---|---|---|---|---|
| 0.0119 | down | -1.7148 | PCYOX1L | prenylcysteine oxidase 1 like |
| 0.0249 | down | -1.7156 | FAM164C | family with sequence similarity 164 member C  transcript variant 2 |
| 0.0290 | down | -1.7157 | GALT | galactose-1-phosphate uridylyltransferase |
| 0.0104 | down | -1.7163 | COX10 | COX10 homolog cytochrome c oxidase assembly protein heme A: farnesyltransferase   nuclear gene encoding mitochondrial protein |
| 0.0010 | down | -1.7188 | RPA3 | replication protein A3 14kDa |
| 0.0344 | down | -1.7193 | PPAPDC2 | phosphatidic acid phosphatase type 2 domain containing 2 |
| 0.0081 | down | -1.7198 | PIGP | phosphatidylinositol glycan anchor biosynthesis class P  transcript variant 2 |
| 0.0048 | down | -1.7205 | C10orf97 | chromosome 10 open reading frame 97 |
| 0.0372 | down | -1.7205 | MED4 | mediator complex subunit 4 |
| 0.0320 | down | -1.7216 | PPIC | peptidylprolyl isomerase C |
| 0.0188 | down | -1.7218 | MRPL35 | mitochondrial ribosomal protein L35  nuclear gene encoding mitochondrial protein transcript variant 1 |
| 0.0332 | down | -1.7230 | LOC730534 | PREDICTED:  similar to D-PCa-2 protein isoform c |
| 0.0076 | down | -1.7243 | TTC27 | tetratricopeptide repeat domain 27 |
| 0.0472 | down | -1.7247 | LOC653506 | PREDICTED:  similar to meteorin glial cell differentiation regulator-like |
| 0.0121 | down | -1.7250 | C14orf108 | chromosome 14 open reading frame 108 |
| 0.0066 | down | -1.7273 | PTCD2 | pentatricopeptide repeat domain 2 |
| 0.0056 | down | -1.7274 | NDUFB3 | NADH dehydrogenase  1 beta subcomplex 3 12kDa |
| 0.0160 | down | -1.7277 | SQLE | squalene epoxidase |
| 0.0314 | down | -1.7282 | KIAA0746 | KIAA0746 protein |
| 0.0181 | down | -1.7284 | ATOX1 | ATX1 antioxidant protein 1 homolog |
| 0.0307 | down | -1.7285 | OGDH | oxoglutarate  dehydrogenase   nuclear gene encoding mitochondrial protein transcript variant 1 |
| 0.0185 | down | -1.7288 | LOC642897 | PREDICTED:  misc_RNA  miscRNA |
| 0.0118 | down | -1.7289 | FLJ20125 | hypothetical protein FLJ20125 |
| 0.0252 | down | -1.7290 | ACO2 | aconitase 2 mitochondrial  nuclear gene encoding mitochondrial protein |
| 0.0252 | down | -1.7302 | ZBED5 | zinc finger BED-type containing 5 |
| 0.0115 | down | -1.7303 | OPLAH | 5-oxoprolinase |
| 0.0204 | down | -1.7332 | ALG8 | asparagine-linked glycosylation 8 homolog   transcript variant 2 |
| 0.0317 | down | -1.7340 | WDR36 | WD repeat domain 36 |
| 0.0008 | down | -1.7378 | TMEM5 | transmembrane protein 5 |
| 0.0359 | down | -1.7385 | MPPE1 | metallophosphoesterase 1 |
| 0.0162 | down | -1.7387 | SRM | spermidine synthase |
| 0.0039 | down | -1.7390 | LANCL1 | LanC lantibiotic synthetase component C-like 1 |
| 0.0206 | down | -1.7392 | NR1H3 | nuclear receptor subfamily 1 group H member 3 |
| 0.0137 | down | -1.7405 | RWDD4A | RWD domain containing 4A |
| 0.0068 | down | -1.7408 | TERF1 | telomeric repeat binding factor  1  transcript variant 1 |
| 0.0162 | down | -1.7409 | ALDH7A1 | aldehyde dehydrogenase 7 family member A1 |
| 0.0370 | down | -1.7410 | ATG4C | ATG4 autophagy related 4 homolog C   transcript variant 7 |
| 0.0374 | down | -1.7417 | FAM111A | family with sequence similarity 111 member A  transcript variant 1 |
| 0.0009 | down | -1.7424 | FH | fumarate hydratase  nuclear gene encoding mitochondrial protein |
| 0.0071 | down | -1.7430 | ETFDH | electron-transferring-flavoprotein dehydrogenase  nuclear gene encoding mitochondrial protein |
| 0.0263 | down | -1.7452 | C7orf36 | chromosome 7 open reading frame 36 |
| 0.0452 | down | -1.7464 | DOLK | dolichol kinase |
| 0.0360 | down | -1.7478 | SMARCAD1 | SWI/SNF-related matrix-associated actin-dependent regulator of chromatin subfamily a containing DEAD/H box 1 |
| 0.0254 | down | -1.7481 | MED20 | mediator complex subunit 20 |
| 0.0108 | down | -1.7486 | UBLCP1 | ubiquitin-like domain containing CTD phosphatase 1 |
| 0.0317 | down | -1.7493 | ZMYM6 | zinc finger MYM-type 6 |
| 0.0471 | down | -1.7505 | ALDH5A1 | aldehyde dehydrogenase 5 family member A1  nuclear gene encoding mitochondrial protein transcript variant 2 |

| | | | | |
|---|---|---|---|---|
| 0.0014 | down | -1.7506 | PTGES2 | prostaglandin E synthase 2 transcript variant 2 |
| 0.0312 | down | -1.7507 | APPBP2 | amyloid beta precursor protein binding protein 2 |
| 0.0337 | down | -1.7512 | C2orf33 | chromosome 2 open reading frame 33 |
| 0.0425 | down | -1.7519 | HNRPH3 | heterogeneous nuclear ribonucleoprotein H3 transcript variant 2H9 |
| 0.0428 | down | -1.7530 | PARP4 | poly polymerase family member 4 |
| 0.0015 | down | -1.7546 | LYRM5 | LYR motif containing 5 |
| 0.0465 | down | -1.7558 | ZMYM4 | zinc finger MYM-type 4 |
| 0.0229 | down | -1.7562 | NHP2L1 | NHP2 non-histone chromosome protein 2-like 1 transcript variant 2 |
| 0.0016 | down | -1.7578 | LYPLAL1 | lysophospholipase-like 1 |
| 0.0018 | down | -1.7603 | PPCS | phosphopantothenoylcysteine synthetase transcript variant 2 |
| 0.0034 | down | -1.7626 | SMC4 | structural maintenance of chromosomes 4 transcript variant 2 |
| 0.0265 | down | -1.7627 | MUTYH | mutY homolog transcript variant gamma2 |
| 0.0386 | down | -1.7640 | NUDT12 | nudix -type motif 12 |
| 0.0198 | down | -1.7644 | RAD51C | RAD51 homolog C transcript variant 1 |
| 0.0078 | down | -1.7653 | OPRS1 | opioid receptor sigma 1 transcript variant 1 |
| 0.0381 | down | -1.7682 | HNRNPA0 | heterogeneous nuclear ribonucleoprotein A0 |
| 0.0083 | down | -1.7690 | UBE2L6 | ubiquitin-conjugating enzyme E2L 6 transcript variant 1 |
| 0.0361 | down | -1.7695 | RNF14 | ring finger protein 14 transcript variant 3 |
| 0.0074 | down | -1.7701 | HSPE1 | heat shock 10kDa protein 1 |
| 0.0059 | down | -1.7708 | C11orf46 | chromosome 11 open reading frame 46 |
| 0.0381 | down | -1.7711 | HMBS | hydroxymethylbilane synthase transcript variant 1 |
| 0.0173 | down | -1.7722 | SLC39A3 | solute carrier family 39 member 3 transcript variant 1 |
| 0.0089 | down | -1.7723 | ORC5L | origin recognition complex subunit 5-like transcript variant 1 |
| 0.0251 | down | -1.7724 | TMEM50B | transmembrane protein 50B |
| 0.0484 | down | -1.7733 | PRICKLE4 | prickle homolog 4 |
| 0.0202 | down | -1.7740 | LOC728635 | PREDICTED: similar to peroxisomal short-chain alcohol dehydrogenase transcript variant 1 |
| 0.0338 | down | -1.7741 | ACACA | acetyl-Coenzyme A carboxylase alpha transcript variant 3 |
| 0.0002 | down | -1.7749 | SDHD | succinate dehydrogenase complex subunit D integral membrane protein nuclear gene encoding mitochondrial protein |
| 0.0266 | down | -1.7751 | RBPMS2 | RNA binding protein with multiple splicing 2 |
| 0.0373 | down | -1.7762 | C14orf142 | chromosome 14 open reading frame 142 |
| 0.0154 | down | -1.7769 | IFI35 | interferon-induced protein 35 |
| 0.0333 | down | -1.7786 | ZNF706 | zinc finger protein 706 transcript variant 3 |
| 0.0129 | down | -1.7808 | RRS1 | RRS1 ribosome biogenesis regulator homolog |
| 0.0377 | down | -1.7826 | PVALB | parvalbumin |
| 0.0068 | down | -1.7826 | C4orf27 | chromosome 4 open reading frame 27 |
| 0.0011 | down | -1.7827 | RPA2 | replication protein A2 32kDa |
| 0.0253 | down | -1.7828 | MTAP | methylthioadenosine phosphorylase |
| 0.0265 | down | -1.7838 | ZC3HAV1 | zinc finger CCCH-type antiviral 1 transcript variant 1 |
| 0.0370 | down | -1.7842 | TMEM126A | transmembrane protein 126A |
| 0.0378 | down | -1.7861 | C11orf17 | chromosome 11 open reading frame 17 transcript variant 1 |
| 0.0407 | down | -1.7870 | CTSC | cathepsin C transcript variant 1 |
| 0.0026 | down | -1.7875 | MRPL3 | mitochondrial ribosomal protein L3 nuclear gene encoding mitochondrial protein |
| 0.0334 | down | -1.7885 | BCAS4 | breast carcinoma amplified sequence 4 transcript variant 1 |
| 0.0088 | down | -1.7893 | RPL34 | ribosomal protein L34 transcript variant 2 |
| 0.0301 | down | -1.7900 | TBC1D7 | TBC1 domain family member 7 |
| 0.0424 | down | -1.7909 | GLCE | glucuronic acid epimerase |
| 0.0465 | down | -1.7916 | LOC401397 | PREDICTED: hypothetical LOC401397 |

| | | | | |
|---|---|---|---|---|
| 0.0239 | down | -1.7944 | LOC148915 | PREDICTED:  similar to Nonhistone chromosomal protein HMG-17 |
| 0.0115 | down | -1.7946 | BCS1L | BCS1-like   nuclear gene encoding mitochondrial protein transcript variant 2 |
| 0.0141 | down | -1.7946 | NFYC | nuclear transcription factor Y gamma |
| 0.0033 | down | -1.7978 | NDUFB3 | NADH dehydrogenase  1 beta subcomplex 3 12kDa |
| 0.0133 | down | -1.7989 | ACADM | acyl-Coenzyme A dehydrogenase C-4 to C-12 straight chain  nuclear gene encoding mitochondrial protein |
| 0.0123 | down | -1.7998 | CCDC132 | coiled-coil domain containing 132  transcript variant 1 |
| 0.0471 | down | -1.8020 | TOMM40L | translocase of outer mitochondrial membrane 40 homolog -like  nuclear gene encoding mitochondrial protein |
| 0.0059 | down | -1.8038 | ADAT1 | adenosine deaminase tRNA-specific 1 |
| 0.0462 | down | -1.8047 | PRMT6 | protein arginine methyltransferase 6 |
| 0.0387 | down | -1.8048 | SFT2D1 | SFT2 domain containing 1 |
| 0.0020 | down | -1.8049 | PPCS | phosphopantothenoylcysteine synthetase  transcript variant 1 |
| 0.0306 | down | -1.8051 | DHRS4 | dehydrogenase/reductase  member 4 |
| 0.0022 | down | -1.8051 | POLR2G | polymerase  II  polypeptide G |
| 0.0096 | down | -1.8056 | AIFM1 | apoptosis-inducing factor mitochondrion-associated 1  nuclear gene encoding mitochondrial protein transcript variant 1 |
| 0.0133 | down | -1.8060 | VPS45 | vacuolar protein sorting 45 homolog |
| 0.0151 | down | -1.8065 | MYO10 | myosin X |
| 0.0357 | down | -1.8067 | PHOSPHO2 | phosphatase orphan 2 |
| 0.0030 | down | -1.8069 | PSMA5 | proteasome  subunit alpha type 5 |
| 0.0187 | down | -1.8071 | NDUFB6 | NADH dehydrogenase  1 beta subcomplex 6 17kDa  nuclear gene encoding mitochondrial protein transcript variant 1 |
| 0.0262 | down | -1.8077 | DHRS7B | dehydrogenase/reductase  member 7B |
| 0.0287 | down | -1.8097 | MRPS27 | mitochondrial ribosomal protein S27  nuclear gene encoding mitochondrial protein |
| 0.0041 | down | -1.8104 | SFXN4 | sideroflexin 4 |
| 0.0038 | down | -1.8113 | ACYP1 | acylphosphatase 1 erythrocyte  type  transcript variant 1 |
| 0.0020 | down | -1.8114 | NDUFS7 | NADH dehydrogenase  Fe-S protein 7 20kDa |
| 0.0412 | down | -1.8120 | RNASEL | ribonuclease L |
| 0.0487 | down | -1.8121 | LOC90624 | hypothetical protein LOC90624 |
| 0.0116 | down | -1.8129 | NSL1 | NSL1 MIND kinetochore complex component homolog   transcript variant 2 |
| 0.0282 | down | -1.8137 | ZNF706 | zinc finger protein 706  transcript variant 2 |
| 0.0231 | down | -1.8138 | SQLE | squalene epoxidase |
| 0.0261 | down | -1.8140 | TTC15 | tetratricopeptide repeat domain 15 |
| 0.0296 | down | -1.8142 | ELOVL5 | ELOVL family member 5 elongation of long chain fatty acids |
| 0.0375 | down | -1.8156 | NME1 | non-metastatic cells 1 protein  expressed in  transcript variant 2 |
| 0.0155 | down | -1.8157 | NUDT5 | nudix -type motif 5 |
| 0.0428 | down | -1.8161 | ZNF260 | zinc finger protein 260 |
| 0.0072 | down | -1.8168 | MRPL24 | mitochondrial ribosomal protein L24  nuclear gene encoding mitochondrial protein transcript variant 2 |
| 0.0390 | down | -1.8176 | MRPS36 | mitochondrial ribosomal protein S36  nuclear gene encoding mitochondrial protein |
| 0.0073 | down | -1.8177 | FLJ20489 | hypothetical protein FLJ20489 |
| 0.0037 | down | -1.8188 | FAH | fumarylacetoacetate hydrolase |
| 0.0166 | down | -1.8191 | CRIPT | cysteine-rich PDZ-binding protein |
| 0.0334 | down | -1.8193 | TMEM103 | transmembrane protein 103 |
| 0.0193 | down | -1.8201 | STEAP2 | six transmembrane epithelial antigen of the prostate 2  transcript variant 1 |
| 0.0229 | down | -1.8205 | GLMN | glomulin FKBP associated protein |
| 0.0339 | down | -1.8238 | MGC4172 | short-chain dehydrogenase/reductase |
| 0.0060 | down | -1.8274 | PSMB8 | proteasome  subunit beta type 8   transcript variant 2 |
| 0.0073 | down | -1.8303 | NUP37 | nucleoporin 37kDa |
| 0.0269 | down | -1.8304 | MRPL35 | mitochondrial ribosomal protein L35  nuclear gene encoding mitochondrial protein transcript variant 1 |
| 0.0358 | down | -1.8321 | HMGN2 | high-mobility group nucleosomal binding domain 2 |

| | | | | |
|---|---|---|---|---|
| 0.0377 | down | -1.8325 | APIP | APAF1 interacting protein |
| 0.0315 | down | -1.8330 | DYNLL1 | dynein light chain LC8-type 1  transcript variant 1 |
| 0.0024 | down | -1.8342 | UROS | uroporphyrinogen III synthase |
| 0.0314 | down | -1.8348 | STAMBP | STAM binding protein  transcript variant 1 |
| 0.0003 | down | -1.8349 | RABEPK | Rab9 effector protein with kelch motifs |
| 0.0031 | down | -1.8349 | C9orf119 | chromosome 9 open reading frame 119 |
| 0.0188 | down | -1.8371 | TSGA14 | testis specific 14 |
| 0.0369 | down | -1.8371 | ORC3L | origin recognition complex subunit 3-like   transcript variant 1 |
| 0.0493 | down | -1.8372 | PFAS | phosphoribosylformylglycinamidine synthase |
| 0.0219 | down | -1.8378 | MBP | myelin basic protein  transcript variant 8 |
| 0.0486 | down | -1.8394 | IPO11 | importin 11 |
| 0.0452 | down | -1.8405 | ELP3 | elongation protein 3 homolog |
| 0.0166 | down | -1.8410 | TRMT5 | TRM5 tRNA methyltransferase 5 homolog |
| 0.0051 | down | -1.8421 | AKR1A1 | aldo-keto reductase family 1 member A1   transcript variant 1 |
| 0.0365 | down | -1.8451 | ANKRD33 | ankyrin repeat domain 33 |
| 0.0122 | down | -1.8451 | ZC3HC1 | zinc finger C3HC-type containing 1 |
| 0.0014 | down | -1.8467 | BOLA3 | bolA homolog 3   transcript variant 1 |
| 0.0253 | down | -1.8476 | PSPH | phosphoserine phosphatase |
| 0.0229 | down | -1.8480 | MYBPC2 | myosin binding protein C fast type |
| 0.0222 | down | -1.8519 | DARS2 | aspartyl-tRNA synthetase 2 mitochondrial  nuclear gene encoding mitochondrial protein |
| 0.0489 | down | -1.8525 | PPIC | peptidylprolyl isomerase C |
| 0.0347 | down | -1.8539 | HSPH1 | heat shock 105kDa/110kDa protein 1 |
| 0.0337 | down | -1.8540 | HNRPH3 | heterogeneous nuclear ribonucleoprotein H3   transcript variant 2H9A |
| 0.0050 | down | -1.8544 | DDX10 | DEAD  box polypeptide 10 |
| 0.0280 | down | -1.8553 | COQ2 | coenzyme Q2 homolog prenyltransferase |
| 0.0102 | down | -1.8580 | IFI30 | interferon gamma-inducible protein 30 |
| 0.0279 | down | -1.8582 | ADSL | adenylosuccinate lyase |
| 0.0054 | down | -1.8592 | AKR1A1 | aldo-keto reductase family 1 member A1   transcript variant 2 |
| 0.0035 | down | -1.8592 | LYPLAL1 | lysophospholipase-like 1 |
| 0.0159 | down | -1.8594 | TMEM55A | transmembrane protein 55A |
| 0.0418 | down | -1.8606 | MGST2 | microsomal glutathione S-transferase 2 |
| 0.0086 | down | -1.8615 | GLRX2 | glutaredoxin 2  transcript variant 1 |
| 0.0069 | down | -1.8622 | LSM2 | LSM2 homolog U6 small nuclear RNA associated |
| 0.0015 | down | -1.8642 | STAT1 | signal transducer and activator of transcription 1 91kDa  transcript variant alpha |
| 0.0155 | down | -1.8643 | AASDHPPT | aminoadipate-semialdehyde dehydrogenase-phosphopantetheinyl transferase |
| 0.0009 | down | -1.8655 | BOLA3 | bolA homolog 3   transcript variant 2 |
| 0.0147 | down | -1.8679 | ILDR1 | immunoglobulin-like domain containing receptor 1 |
| 0.0162 | down | -1.8690 | APOO | apolipoprotein O |
| 0.0288 | down | -1.8699 | RTN4IP1 | reticulon 4 interacting protein 1  nuclear gene encoding mitochondrial protein |
| 0.0388 | down | -1.8706 | COMMD8 | COMM domain containing 8 |
| 0.0310 | down | -1.8757 | RRP15 | ribosomal RNA processing 15 homolog |
| 0.0118 | down | -1.8763 | TSPAN13 | tetraspanin 13 |
| 0.0174 | down | -1.8773 | LAP3 | leucine aminopeptidase 3 |
| 0.0315 | down | -1.8872 | SLC35A5 | solute carrier family 35 member A5 |
| 0.0411 | down | -1.8872 | TTC31 | tetratricopeptide repeat domain 31 |
| 0.0332 | down | -1.8876 | NDUFB2 | NADH dehydrogenase  1 beta subcomplex 2 8kDa  nuclear gene encoding mitochondrial protein |
| 0.0155 | down | -1.8880 | KIAA0528 | KIAA0528 |

| | | | | |
|---|---|---|---|---|
| 0.0145 | down | -1.8881 | GEMIN6 | gem  associated protein 6 |
| 0.0001 | down | -1.8882 | C7orf50 | chromosome 7 open reading frame 50 |
| 0.0112 | down | -1.8888 | GCA | grancalcin EF-hand calcium binding protein |
| 0.0258 | down | -1.8899 | FANCL | Fanconi anemia complementation group L |
| 0.0020 | down | -1.8899 | ACAT1 | acetyl-Coenzyme A acetyltransferase 1   nuclear gene encoding mitochondrial protein |
| 0.0469 | down | -1.8923 | KLHDC9 | kelch domain containing 9  transcript variant 3 |
| 0.0027 | down | -1.8929 | TATDN3 | TatD DNase domain containing 3  transcript variant 1 |
| 0.0143 | down | -1.8948 | DCN | decorin  transcript variant C |
| 0.0206 | down | -1.8967 | CCDC56 | coiled-coil domain containing 56 |
| 0.0414 | down | -1.8981 | MCM6 | minichromosome maintenance complex component 6 |
| 0.0435 | down | -1.8984 | CTSB | cathepsin B  transcript variant 2 |
| 0.0039 | down | -1.9002 | HSPA8 | heat shock 70kDa protein 8  transcript variant 1 |
| 0.0000 | down | -1.9006 | MRPS33 | mitochondrial ribosomal protein S33  nuclear gene encoding mitochondrial protein transcript variant 1 |
| 0.0145 | down | -1.9009 | C14orf108 | chromosome 14 open reading frame 108 |
| 0.0202 | down | -1.9016 | GART | phosphoribosylglycinamide formyltransferase phosphoribosylglycinamide synthetase phosphoribosylaminoimidazole synthetase  transcript variant 1 |
| 0.0041 | down | -1.9024 | HSPA8 | heat shock 70kDa protein 8  transcript variant 2 |
| 0.0332 | down | -1.9029 | HDHD2 | haloacid dehalogenase-like hydrolase domain containing 2 |
| 0.0421 | down | -1.9039 | OAF | OAF homolog |
| 0.0413 | down | -1.9040 | APITD1 | apoptosis-inducing TAF9-like domain 1  transcript variant B |
| 0.0407 | down | -1.9050 | CKLF | chemokine-like factor  transcript variant 6 |
| 0.0166 | down | -1.9058 | TMEM126B | transmembrane protein 126B |
| 0.0173 | down | -1.9067 | FDX1L | ferredoxin 1-like |
| 0.0121 | down | -1.9077 | SRBD1 | S1 RNA binding domain 1 |
| 0.0285 | down | -1.9077 | MFSD3 | major facilitator superfamily domain containing 3 |
| 0.0049 | down | -1.9101 | LGMN | legumain  transcript variant 2 |
| 0.0032 | down | -1.9121 | DIMT1L | DIM1 dimethyladenosine transferase 1-like |
| 0.0409 | down | -1.9162 | H2AFY2 | H2A histone family member Y2 |
| 0.0057 | down | -1.9183 | DECR1 | 24-dienoyl CoA reductase 1 mitochondrial  nuclear gene encoding mitochondrial protein |
| 0.0352 | down | -1.9191 | STEAP2 | six transmembrane epithelial antigen of the prostate 2 |
| 0.0106 | down | -1.9214 | PARP9 | poly  polymerase family member 9 |
| 0.0447 | down | -1.9238 | C1orf19 | chromosome 1 open reading frame 19 |
| 0.0016 | down | -1.9240 | PRMT3 | protein arginine methyltransferase 3 |
| 0.0117 | down | -1.9242 | PRDX6 | peroxiredoxin 6 |
| 0.0205 | down | -1.9264 | KIAA0391 | KIAA0391 |
| 0.0343 | down | -1.9282 | SUOX | sulfite oxidase  nuclear gene encoding mitochondrial protein transcript variant 2 |
| 0.0216 | down | -1.9299 | ACADM | acyl-Coenzyme A dehydrogenase C-4 to C-12 straight chain  nuclear gene encoding mitochondrial protein |
| 0.0115 | down | -1.9309 | PTRH1 | peptidyl-tRNA hydrolase 1 homolog |
| 0.0298 | down | -1.9317 | KLHDC5 | kelch domain containing 5 |
| 0.0135 | down | -1.9338 | XTP3TPA | XTP3-transactivated protein A |
| 0.0431 | down | -1.9361 | EXTL2 | exostoses -like 2  transcript variant 1 |
| 0.0244 | down | -1.9379 | TMEM42 | transmembrane protein 42 |
| 0.0286 | down | -1.9410 | FAHD2B | fumarylacetoacetate hydrolase domain containing 2B |
| 0.0026 | down | -1.9413 | LOC729101 | PREDICTED:  misc_RNA  miscRNA |
| 0.0346 | down | -1.9416 | C1orf156 | chromosome 1 open reading frame 156 |
| 0.0181 | down | -1.9433 | FAM108C1 | family with sequence similarity 108 member C1 |
| 0.0275 | down | -1.9435 | HNMT | histamine N-methyltransferase  transcript variant 1 |
| 0.0139 | down | -1.9514 | PCK2 | phosphoenolpyruvate carboxykinase 2   nuclear gene encoding mitochondrial protein transcript variant 1 |

| | | | | |
|---|---|---|---|---|
| 0.0074 | down | -1.9538 | C18orf55 | chromosome 18 open reading frame 55 |
| 0.0110 | down | -1.9561 | ADSS | adenylosuccinate synthase |
| 0.0396 | down | -1.9565 | IDH1 | isocitrate dehydrogenase 1 soluble |
| 0.0064 | down | -1.9634 | ABCC3 | ATP-binding cassette sub-family C member 3 |
| 0.0120 | down | -1.9636 | CCND1 | cyclin D1 |
| 0.0097 | down | -1.9664 | LOC113386 | similar to envelope protein |
| 0.0054 | down | -1.9670 | MRPS35 | mitochondrial ribosomal protein S35 nuclear gene encoding mitochondrial protein |
| 0.0388 | down | -1.9680 | CTPS | CTP synthase |
| 0.0491 | down | -1.9707 | WASPIP | Wiskott-Aldrich syndrome protein interacting protein |
| 0.0118 | down | -1.9713 | RAD51C | RAD51 homolog C transcript variant 1 |
| 0.0111 | down | -1.9715 | APEH | N-acylaminoacyl-peptide hydrolase |
| 0.0015 | down | -1.9736 | MRPL15 | mitochondrial ribosomal protein L15 nuclear gene encoding mitochondrial protein |
| 0.0002 | down | -1.9757 | HSPE1 | heat shock 10kDa protein 1 |
| 0.0128 | down | -1.9760 | WDR12 | WD repeat domain 12 |
| 0.0227 | down | -1.9762 | NDUFB5 | NADH dehydrogenase 1 beta subcomplex 5 16kDa nuclear gene encoding mitochondrial protein |
| 0.0054 | down | -1.9766 | ARV1 | ARV1 homolog |
| 0.0146 | down | -1.9829 | NDUFA3 | NADH dehydrogenase 1 alpha subcomplex 3 9kDa |
| 0.0079 | down | -1.9839 | TMEM126B | transmembrane protein 126B |
| 0.0020 | down | -1.9867 | ATIC | 5-aminoimidazole-4-carboxamide ribonucleotide formyltransferase/IMP cyclohydrolase |
| 0.0268 | down | -1.9875 | HERC5 | hect domain and RLD 5 |
| 0.0013 | down | -1.9896 | STAT1 | signal transducer and activator of transcription 1 91kDa transcript variant beta |
| 0.0207 | down | -1.9904 | PPARGC1A | peroxisome proliferator-activated receptor gamma coactivator 1 alpha |
| 0.0043 | down | -1.9950 | DCI | dodecenoyl-Coenzyme A delta isomerase nuclear gene encoding mitochondrial protein |
| 0.0365 | down | -1.9954 | OMA1 | OMA1 homolog zinc metallopeptidase |
| 0.0333 | down | -1.9958 | CYP27A1 | cytochrome P450 family 27 subfamily A polypeptide 1 nuclear gene encoding mitochondrial protein |
| 0.0010 | down | -1.9958 | LACTB2 | lactamase beta 2 |
| 0.0444 | down | -1.9984 | NUP43 | nucleoporin 43kDa transcript variant 2 |
| 0.0362 | down | -1.9989 | TSEN2 | tRNA splicing endonuclease 2 homolog |
| 0.0126 | down | -2.0038 | C9orf23 | chromosome 9 open reading frame 23 transcript variant 1 |
| 0.0130 | down | -2.0046 | ECHDC1 | enoyl Coenzyme A hydratase domain containing 1 |
| 0.0099 | down | -2.0046 | POLR3GL | polymerase III polypeptide G -like |
| 0.0336 | down | -2.0080 | LEPROTL1 | leptin receptor overlapping transcript-like 1 |
| 0.0086 | down | -2.0104 | TMEM218 | transmembrane protein 218 |
| 0.0269 | down | -2.0109 | POP5 | processing of precursor 5 ribonuclease P/MRP subunit transcript variant 3 |
| 0.0177 | down | -2.0116 | DISP1 | dispatched homolog 1 |
| 0.0119 | down | -2.0119 | PIGF | phosphatidylinositol glycan anchor biosynthesis class F transcript variant 1 |
| 0.0381 | down | -2.0145 | DNAJC19 | DnaJ homolog subfamily C member 19 |
| 0.0153 | down | -2.0194 | ALG6 | asparagine-linked glycosylation 6 homolog |
| 0.0030 | down | -2.0279 | C11orf1 | chromosome 11 open reading frame 1 |
| 0.0424 | down | -2.0312 | ACY1 | aminoacylase 1 |
| 0.0106 | down | -2.0332 | WDR12 | WD repeat domain 12 |
| 0.0022 | down | -2.0338 | MRPL24 | mitochondrial ribosomal protein L24 nuclear gene encoding mitochondrial protein transcript variant 1 |
| 0.0320 | down | -2.0355 | ACACB | acetyl-Coenzyme A carboxylase beta |
| 0.0093 | down | -2.0367 | C1QC | complement component 1 q subcomponent C chain |
| 0.0109 | down | -2.0371 | HADH | hydroxyacyl-Coenzyme A dehydrogenase nuclear gene encoding mitochondrial protein |
| 0.0134 | down | -2.0372 | HSD17B8 | hydroxysteroid dehydrogenase 8 |
| 0.0452 | down | -2.0427 | RAB23 | RAB23 member RAS oncogene family transcript variant 1 |

| | | | | |
|---|---|---|---|---|
| 0.0306 | down | -2.0456 | ATP5G3 | ATP synthase H+ transporting mitochondrial F0 complex subunit C3   nuclear gene encoding mitochondrial protein transcript variant 3 |
| 0.0036 | down | -2.0462 | NQO1 | NADH dehydrogenase quinone 1  transcript variant 1 |
| 0.0001 | down | -2.0494 | TIMM10 | translocase of inner mitochondrial membrane 10 homolog   nuclear gene encoding mitochondrial protein |
| 0.0199 | down | -2.0501 | CKLF | chemokine-like factor  transcript variant 5 |
| 0.0187 | down | -2.0504 | NNT | nicotinamide nucleotide transhydrogenase |
| 0.0477 | down | -2.0507 | PIR | pirin   transcript variant 2 |
| 0.0245 | down | -2.0524 | PSIP1 | PC4 and SFRS1 interacting protein 1  transcript variant 2 |
| 0.0128 | down | -2.0560 | APTX | aprataxin  transcript variant 2 |
| 0.0101 | down | -2.0572 | HMGCR | 3-hydroxy-3-methylglutaryl-Coenzyme A reductase |
| 0.0231 | down | -2.0584 | BRI3BP | PREDICTED:  BRI3 binding protein |
| 0.0399 | down | -2.0587 | ZMYND15 | zinc finger MYND-type containing 15 |
| 0.0085 | down | -2.0593 | SFXN4 | sideroflexin 4  transcript variant 3 |
| 0.0387 | down | -2.0608 | GNPDA1 | glucosamine-6-phosphate deaminase 1 |
| 0.0064 | down | -2.0619 | C9orf46 | chromosome 9 open reading frame 46 |
| 0.0136 | down | -2.0640 | C1QB | complement component 1 q subcomponent B chain |
| 0.0421 | down | -2.0683 | MRPL52 | mitochondrial ribosomal protein L52  nuclear gene encoding mitochondrial protein transcript variant 6 |
| 0.0172 | down | -2.0721 | | cDNA clone IMAGE:5277162 |
| 0.0195 | down | -2.0735 | SPG11 | spastic paraplegia 11 |
| 0.0182 | down | -2.0767 | POLR3B | polymerase  III  polypeptide B |
| 0.0117 | down | -2.0865 | ASF1A | ASF1 anti-silencing function 1 homolog A |
| 0.0066 | down | -2.0868 | SDAD1 | SDA1 domain containing 1 |
| 0.0146 | down | -2.0913 | ATP5L | ATP synthase H+ transporting mitochondrial F0 complex subunit G  nuclear gene encoding mitochondrial protein |
| 0.0365 | down | -2.0924 | MGST1 | microsomal glutathione S-transferase 1  transcript variant 1a |
| 0.0368 | down | -2.0929 | MRPL52 | mitochondrial ribosomal protein L52  nuclear gene encoding mitochondrial protein transcript variant 2 |
| 0.0140 | down | -2.0931 | HMGCS1 | 3-hydroxy-3-methylglutaryl-Coenzyme A synthase 1   transcript variant 2 |
| 0.0208 | down | -2.0933 | PPIL1 | peptidylprolyl isomerase -like 1 |
| 0.0213 | down | -2.0958 | NBN | nibrin |
| 0.0390 | down | -2.0969 | LYSMD2 | LysM putative peptidoglycan-binding domain containing 2 |
| 0.0301 | down | -2.0973 | FAM111A | family with sequence similarity 111 member A  transcript variant 1 |
| 0.0002 | down | -2.0985 | NQO1 | NADH dehydrogenase quinone 1  transcript variant 1 |
| 0.0259 | down | -2.1024 | NIPSNAP1 | nipsnap homolog 1 |
| 0.0013 | down | -2.1123 | TALDO1 | transaldolase 1 |
| 0.0213 | down | -2.1192 | NNT | nicotinamide nucleotide transhydrogenase  nuclear gene encoding mitochondrial protein transcript variant 1 |
| 0.0019 | down | -2.1198 | ZNF331 | zinc finger protein 331  transcript variant 3 |
| 0.0455 | down | -2.1199 | TMEM150 | transmembrane protein 150  transcript variant 1 |
| 0.0443 | down | -2.1201 | LOC124220 | similar to common salivary protein 1 |
| 0.0179 | down | -2.1279 | CYP4V2 | cytochrome P450 family 4 subfamily V polypeptide 2 |
| 0.0235 | down | -2.1342 | SC4MOL | sterol-C4-methyl oxidase-like  transcript variant 1 |
| 0.0490 | down | -2.1353 | WDR68 | WD repeat domain 68 |
| 0.0466 | down | -2.1363 | MCM3 | minichromosome maintenance complex component 3 |
| 0.0499 | down | -2.1392 | ZNF302 | zinc finger protein 302  transcript variant 1 |
| 0.0210 | down | -2.1408 | WDR4 | WD repeat domain 4  transcript variant 2 |
| 0.0387 | down | -2.1436 | QDPR | quinoid dihydropteridine reductase |
| 0.0393 | down | -2.1442 | RNASEL | ribonuclease L |
| 0.0213 | down | -2.1451 | TM2D2 | TM2 domain containing 2  transcript variant 1 |
| 0.0277 | down | -2.1500 | C17orf97 | chromosome 17 open reading frame 97 |
| 0.0021 | down | -2.1549 | ALDH3A2 | aldehyde dehydrogenase 3 family member A2  transcript variant 1 |

| | | | | |
|---|---|---|---|---|
| 0.0267 | down | -2.1594 | ALDH5A1 | aldehyde dehydrogenase 5 family member A1  nuclear gene encoding mitochondrial protein transcript variant 2 |
| 0.0106 | down | -2.1625 | RPUSD3 | RNA pseudouridylate synthase domain containing 3 |
| 0.0437 | down | -2.1693 | CBY1 | chibby homolog 1   transcript variant 1 |
| 0.0022 | down | -2.1736 | GPX1 | glutathione peroxidase 1  transcript variant 2 |
| 0.0289 | down | -2.1743 | PEX11B | peroxisomal biogenesis factor 11 beta |
| 0.0088 | down | -2.1775 | NSMCE4A | non-SMC element 4 homolog A |
| 0.0237 | down | -2.1824 | ADHFE1 | alcohol dehydrogenase iron containing 1  transcript variant 1 |
| 0.0190 | down | -2.1833 | SIRT5 | sirtuin  5   transcript variant 1 |
| 0.0197 | down | -2.1842 | FUCA1 | fucosidase alpha-L- 1 tissue |
| 0.0212 | down | -2.1911 | NPM3 | nucleophosmin/nucleoplasmin 3 |
| 0.0332 | down | -2.1920 | VPS36 | vacuolar protein sorting 36 homolog |
| 0.0105 | down | -2.2013 | DPH5 | DPH5 homolog   transcript variant 3 |
| 0.0023 | down | -2.2098 | AIFM1 | apoptosis-inducing factor mitochondrion-associated 1  nuclear gene encoding mitochondrial protein transcript variant 3 |
| 0.0121 | down | -2.2128 | GSTK1 | glutathione S-transferase kappa 1 |
| 0.0212 | down | -2.2252 | C6orf108 | chromosome 6 open reading frame 108  transcript variant 2 |
| 0.0387 | down | -2.2280 | BIVM | basic immunoglobulin-like variable motif containing |
| 0.0130 | down | -2.2282 | LARP4 | La ribonucleoprotein domain family member 4  transcript variant 1 |
| 0.0178 | down | -2.2317 | COMMD3 | COMM domain containing 3 |
| 0.0224 | down | -2.2422 | NNT | nicotinamide nucleotide transhydrogenase  nuclear gene encoding mitochondrial protein transcript variant 1 |
| 0.0015 | down | -2.2478 | MRPL35 | mitochondrial ribosomal protein L35  nuclear gene encoding mitochondrial protein transcript variant 2 |
| 0.0386 | down | -2.2602 | CYP1B1 | cytochrome P450 family 1 subfamily B polypeptide 1 |
| 0.0258 | down | -2.2673 | C4orf43 | chromosome 4 open reading frame 43 |
| 0.0312 | down | -2.2696 | SUOX | sulfite oxidase  nuclear gene encoding mitochondrial protein transcript variant 1 |
| 0.0055 | down | -2.2703 | RPP40 | ribonuclease P/MRP 40kDa subunit |
| 0.0332 | down | -2.2792 | MOSC2 | MOCO sulphurase C-terminal domain containing 2 |
| 0.0449 | down | -2.2801 | TRIOBP | TRIO and F-actin binding protein  transcript variant 6 |
| 0.0164 | down | -2.2943 | CMBL | carboxymethylenebutenolidase homolog |
| 0.0400 | down | -2.3023 | TFPI2 | tissue factor pathway inhibitor 2 |
| 0.0219 | down | -2.3046 | ADHFE1 | alcohol dehydrogenase iron containing 1 |
| 0.0329 | down | -2.3192 | NLGN4X | neuroligin 4 X-linked  transcript variant 1 |
| 0.0347 | down | -2.3193 | TFRC | transferrin receptor |
| 0.0449 | down | -2.3207 | OMA1 | OMA1 homolog zinc metallopeptidase |
| 0.0161 | down | -2.3317 | ACSL3 | acyl-CoA synthetase long-chain family member 3  transcript variant 1 |
| 0.0190 | down | -2.3392 | TAF9L | TAF9-like RNA polymerase II TATA box binding protein -associated factor 31kDa |
| 0.0013 | down | -2.3460 | GOT1 | glutamic-oxaloacetic transaminase 1 soluble |
| 0.0075 | down | -2.3543 | ETV5 | ets variant gene 5 |
| 0.0158 | down | -2.3720 | C17orf97 | chromosome 17 open reading frame 97 |
| 0.0064 | down | -2.3787 | MRPL1 | mitochondrial ribosomal protein L1  nuclear gene encoding mitochondrial protein |
| 0.0002 | down | -2.3902 | ACTA2 | actin alpha 2 smooth muscle aorta |
| 0.0026 | down | -2.3942 | PNPO | pyridoxamine 5'-phosphate oxidase |
| 0.0254 | down | -2.4105 | C3orf31 | chromosome 3 open reading frame 31 |
| 0.0007 | down | -2.4182 | PCNA | proliferating cell nuclear antigen  transcript variant 2 |
| 0.0150 | down | -2.4206 | TSGA14 | testis specific 14 |
| 0.0143 | down | -2.4261 | KIAA1618 | KIAA1618 |
| 0.0226 | down | -2.4298 | COQ5 | coenzyme Q5 homolog methyltransferase |
| 0.0194 | down | -2.4363 | HIBCH | 3-hydroxyisobutyryl-Coenzyme A hydrolase  transcript variant 2 |
| 0.0301 | down | -2.4379 | RGS2 | regulator of G-protein signalling 2 24kDa |

| | | | | |
|---|---|---|---|---|
| 0.0045 | down | -2.4509 | STAT1 | signal transducer and activator of transcription 1 91kDa  transcript variant alpha |
| 0.0004 | down | -2.4562 | SDSL | serine dehydratase-like |
| 0.0471 | down | -2.4772 | NPL | N-acetylneuraminate pyruvate lyase |
| 0.0493 | down | -2.4850 | CTPS2 | CTP synthase II  transcript variant 2 |
| 0.0387 | down | -2.5282 | IRX3 | iroquois homeobox 3 |
| 0.0144 | down | -2.5297 | ATP5G1 | ATP synthase H+ transporting mitochondrial F0 complex subunit C1   nuclear gene encoding mitochondrial protein transcript variant 2 |
| 0.0204 | down | -2.5304 | FANCL | Fanconi anemia complementation group L |
| 0.0477 | down | -2.5492 | NPL | N-acetylneuraminate pyruvate lyase |
| 0.0432 | down | -2.6917 | PLA2G7 | phospholipase A2 group VII |
| 0.0023 | down | -2.7111 | ALDH3A2 | aldehyde dehydrogenase 3 family member A2  transcript variant 2 |
| 0.0392 | down | -2.7230 | ESRRG | estrogen-related receptor gamma  transcript variant 2 |
| 0.0067 | down | -2.7238 | THEM2 | thioesterase superfamily member 2 |
| 0.0388 | down | -2.7938 | IDH1 | isocitrate dehydrogenase 1  soluble |
| 0.0222 | down | -3.3111 | SFRS7 | splicing factor arginine/serine-rich 7 35kDa |
| 0.0201 | down | -3.4190 | HLA-DMB | major histocompatibility complex class II DM beta |
| 0.0449 | down | -3.5386 | CCL2 | chemokine  ligand 2 |
